# Antiviral capacity of the early CD8 T-cell response is predictive of natural control of SIV infection: Learning *in vivo* dynamics using *ex vivo* data

**DOI:** 10.1371/journal.pcbi.1012434

**Published:** 2024-09-10

**Authors:** Bharadwaj Vemparala, Vincent Madelain, Caroline Passaes, Antoine Millet, Véronique Avettand-Fenoel, Ramsès Djidjou-Demasse, Nathalie Dereuddre-Bosquet, Roger Le Grand, Christine Rouzioux, Bruno Vaslin, Asier Sáez-Cirión, Jérémie Guedj, Narendra M. Dixit

**Affiliations:** 1 Department of Chemical Engineering, Indian Institute of Science, Bengaluru, India; 2 Université Paris Cité, IAME, INSERM, Paris, France; 3 Institut Pasteur, Université Paris Cité, Viral Reservoirs and Immune Control Unit, Paris, France; 4 CEA, Université Paris-Saclay, INSERM U1184, Immunology of Viral, Autoimmune, Hematologic and Bacterial Diseases (IMVAHB), IDMIT Department/ IBFJ, Fontenay-aux-Roses, France; 5 INSERM U1016, CNRS UMR8104, Université Paris Cité Institut Cochin, Paris, France; 6 MIVEGEC, University of Montpellier, CNRS, IRD, Montpellier, France; 7 Department of Bioengineering, Indian Institute of Science, Bengaluru, India; University of California San Diego Division of Biological Sciences, UNITED STATES OF AMERICA

## Abstract

While most individuals suffer progressive disease following HIV infection, a small fraction spontaneously controls the infection. Although CD8 T-cells have been implicated in this natural control, their mechanistic roles are yet to be established. Here, we combined mathematical modeling and analysis of previously published data from 16 SIV-infected macaques, of which 12 were natural controllers, to elucidate the role of CD8 T-cells in natural control. For each macaque, we considered, in addition to the canonical *in vivo* plasma viral load and SIV DNA data, longitudinal *ex vivo* measurements of the virus suppressive capacity of CD8 T-cells. Available mathematical models do not allow analysis of such combined *in vivo*-*ex vivo* datasets. We explicitly modeled the *ex vivo* assay, derived analytical approximations that link the *ex vivo* measurements with the *in vivo* effector function of CD8-T cells, and integrated them with an *in vivo* model of virus dynamics, thus developing a new learning framework that enabled the analysis. Our model fit the data well and estimated the recruitment rate and/or maximal killing rate of CD8 T-cells to be up to 2-fold higher in controllers than non-controllers (p = 0.013). Importantly, the cumulative suppressive capacity of CD8 T-cells over the first 4–6 weeks of infection was associated with virus control (Spearman’s ρ = -0.51; p = 0.05). Thus, our analysis identified the early cumulative suppressive capacity of CD8 T-cells as a predictor of natural control. Furthermore, simulating a large virtual population, our model quantified the minimum capacity of this early CD8 T-cell response necessary for long-term control. Our study presents new, quantitative insights into the role of CD8 T-cells in the natural control of HIV infection and has implications for remission strategies.

## Author summary

HIV infection results in progressive disease culminating in AIDS in most individuals if left untreated. A rare set of individuals, called natural controllers, however, can control the virus without treatment. Understanding what underlies such control may help devise strategies to elicit it in non-controllers. A challenge has been the limited understanding of the mechanistic role of CD8 T-cells. Here, using new mathematical modeling which enabled simultaneous analysis of *in vivo* and *ex vivo* longitudinal data from macaques infected with SIV, we identified the cumulative infection suppressive capacity of CD8 T-cells early in infection (4–6 weeks) as a measurable correlate of control in the chronic phase of the infection. This cumulative suppressive capacity was significantly higher in controllers than non-controllers, attributed by our model to superior CD8 T-cell recruitment rate and/or maximal killing rate in the controllers. Our study quantifies the role of CD8 T-cells in the natural control of SIV and has implications for interventions targeting long-term HIV remission.

## Introduction

Antiretroviral therapy (ART) suppresses viremia in individuals with HIV and arrests progression to AIDS but does not eradicate the virus [1]. Stopping treatment even after years of HIV control under ART typically results in viral recrudescence and disease progression. ART must therefore be administered lifelong. Enormous efforts are underway to devise interventions that could elicit long-term virus control following short-term drug exposure [2–5]. These efforts are inspired by the rare individuals, termed ‘natural controllers,’ who control viremia without any intervention [6].

Efforts to identify the determinants of natural control, in humans and non-human primates, point to the crucial role of CD8 T-cells in establishing such control. Natural controllers have an over-representation of the protective major histocompatibility complex (MHC) class-I haplotypes, like B*57 and B*27, which appear to facilitate strong, cross-reactive CD8 T-cell responses to HIV [7–9]. Natural controllers tend to have a higher frequency of polyfunctional [9,10] and Gag-specific [11, 12] CD8 T-cells and exhibit lower levels of CD8 T-cell exhaustion [13] than non-controllers. Furthermore, memory-like CD8 T-cells were reported to develop early after infection in controllers [14], which may confer protective immunity. Conversely, suboptimal CD8 T-cell responses were correlated with impaired virus control [13,15,16].

Despite this substantial evidence, the processes determining CD8 T-cell response kinetics that underlie natural control are yet to be clearly elucidated. This is possibly because most studies offer either a static snapshot or a qualitative measure of the CD8 T-cell response, whereas the CD8 T-cell response is dynamic and influences disease outcome by its quality as well as magnitude [17]. Indeed, the frequency of the CD8 T-cells alone was found not to be a reliable indicator of natural control [10,14,18].

In an effort to characterize the CD8 T-cell response more comprehensively, an *ex vivo* assay was developed some years ago [19] and has since been employed in multiple studies on HIV and SIV infections [9,11,14,20–24]. The assay measures the capacity of the CD8 T-cells drawn from an individual to suppress the viral load in a culture of autologous target CD4 T-cells exposed to the virus. This ‘suppressive capacity’ is thus a composite measure of the quality and the quantity of the CD8 T-cells. Furthermore, longitudinal measurements of the suppressive capacity provide a dynamic measure of the CD8 T-cell response during infection and hold promise of elucidating its mechanistic underpinnings in natural control. Because of the complex, nonlinear interactions between CD8 T-cells and antigen, however, identifying characteristics of the CD8 T-cell response associated with virus control would require analysis of the suppressive capacity measurements simultaneously with measurements of plasma viral load and other markers of disease state, such as the frequency of infected cells. Available mathematical models of virus dynamics have yielded profound insights into long-term HIV/SIV control [8,25–28] but are incapable of this analysis. The challenge arises from the multiscale and combined *in vivo-ex vivo* nature of the dataset, which current models cannot handle. Here, we developed a new mathematical model that enables this analysis. We made conceptual advances based on which our model not only described the suppressive capacity measurements but also explicitly incorporated the influence of the suppressive capacity on *in vivo* virus dynamics, enabling learning *in vivo* effector responses. We applied the model to analyze published data from an SIV-cynomolgus macaque model [14], which showed robust maturation of virus-specific CD8 T-cell responses in natural controllers. We found that the cumulative CD8 T-cell suppressive capacity early in the infection was a correlate of natural control at later stages.

## Results

### Model integrating *ex vivo* CD8 T-cell suppressive capacity with *in vivo* virus dynamics

**Fig 1:**
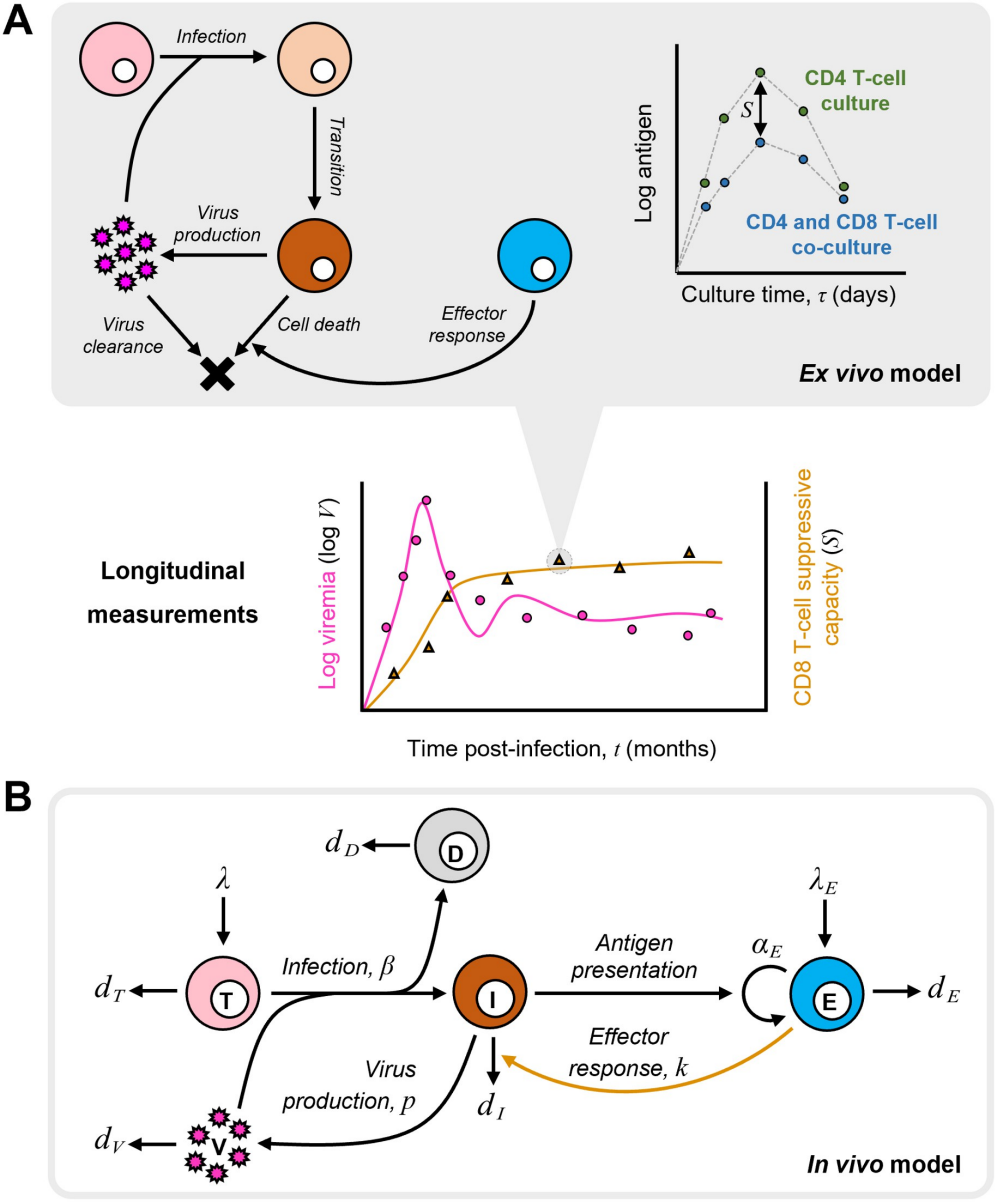
Schematic of the mathematical model. **(A) Model of the *ex vivo* assay.** The events in the *ex vivo* cultures (left) leading to the dynamics (right) and the reported suppressive capacity (*S*) as the difference in the antigen load in the cultures with and without CD8 T-cells. The model enables prediction of *S* and hence analysis of its longitudinal measurements along with *in vivo* measurements such as viremia (bottom), when integrated in a model of *in vivo* dynamics. **(B) Model of *in vivo* dynamics.** The events driving *in vivo* infection contained in our model, including the CD8 T-cell suppressive capacity reflected in the effector response (yellow arrow), linking the *ex vivo* and *in vivo* datasets (Methods).

We developed our model in three stages (Methods): First, we modeled virus dynamics in the *ex vivo* cultures, quantifying the CD8 T-cell suppressive capacity (S1 Fig). Second, we derived analytical expressions from the *ex vivo* model that linked the suppressive capacity with the killing rate of infected cells by CD8 T-cells. Third, we integrated the analytical expressions into a model of *in vivo* virus dynamics, thereby constructing a unified framework capable of simultaneously predicting and hence fitting the measured *in vivo* and *ex vivo* quantities. We tested variants of the *in vivo* model using a formal model building strategy (S1 Text and S2 –S10 Figs and S1–S10 Tables) to identify the best model (Methods). The following equations describe the resulting model (Fig 1)

$$(\text{Target}\;\text{cells})\;\;\;\;\;\frac{dT}{dt}=\lambda -\beta TV-d_{T}T$$ (1)

$$\left(\begin{array}{l} \text{Productively}\\ \text{infected}\;\text{cells} \end{array}\right)\;\;\;\;\;\frac{dI}{dt}=(1-f_{D})\beta TV-kEI-d_{I}I$$ (2)

$$\left(\begin{array}{l} \text{Non-productively}\;\\ \text{infected}\;\text{cells} \end{array}\right)\;\;\;\;\;\frac{dD}{dt}=f_{D}\beta TV-d_{D}D$$ (3)

$$(\text{Viremia})\;\;\;\;\;\frac{dV}{dt}=pI-d_{V}V$$ (4)

$$\left(\begin{array}{l} \text{Effector}\\ \text{CD8}\;\text{T-cells} \end{array}\right)\;\;\;\;\;\frac{dE}{dt}=\lambda_{E}+\alpha_{E}E\frac{I}{\theta_{E}+I}-d_{E}E$$ (5)

$$(\text{Killing}\;\text{rate})\;\;\;\;\;\frac{dk}{dt}=\omega \left(k_{f}-k\right)$$ (6)

$$\left(\begin{array}{l} \text{Suppressive}\\ \text{capacity} \end{array}\right)\;\;\;\;\;S(\sigma )={\text{log}}_{10}\left(\hat{V}\left(0,\tau_{\text{max}}\right)\right)-{\text{log}}_{10}\left(\hat{V}\left(\sigma ,\tau_{\text{max}}\right)\right)$$ (7)

$$\left(\begin{array}{l} Ex\;vivo\\ \text{antigen}\;\text{load} \end{array}\right)\;\;\;\;\;\hat{V}(\sigma ,\tau )=\frac{{\hat{V}}_{0}e^{-\frac{\tau (\sigma +\delta +\rho )}{2}}}{2\alpha }\left(\left(\sigma +\delta -\rho +\alpha \right)e^{-\frac{\tau \alpha }{2}}+\left(\rho -\sigma -\delta +\alpha \right)e^{\frac{\tau \alpha }{2}}\right)$$ (8)

$$\left(\begin{array}{l} \text{Time}\;\text{to}\;\text{peak}\\ \text{antigen}\;\text{load} \end{array}\right)\;\;\;\;\;\tau_{\text{max}}=\frac{2}{\alpha_{n}-\rho }\text{ln}\left[\frac{\alpha_{n}}{{\hat{V}}_{0}\hat{\beta }}\frac{\rho +\alpha_{n}}{\rho -\alpha_{n}}\text{ln}\left(\frac{\rho \delta }{(1-f)\hat{\beta }\phi {\hat{T}}_{0}}\right)\right]$$ (9)

$$\left(\begin{array}{l} Ex\;vivo\\ \text{effector}\;\text{response} \end{array}\right)\;\;\;\;\;\sigma =k{\hat{C}}_{0}\left(\frac{E}{C_{0}}\right)$$ (10)

where $\alpha =\sqrt{{\left(\sigma +\delta -\rho \right)}^{2}+4(1-f)\hat{\beta }{\hat{T}}_{0}\phi }$ and $\alpha_{n}=\sqrt{\rho^{2}+4(1-f)\hat{\beta }{\hat{T}}_{0}\phi }$.

Here, uninfected CD4 T-cells, *T*, are recruited at the rate *λ* and die at the rate *d*_*T*_*T*. They get infected by free virions in plasma, *V*, at the rate *βTV*. Because infected cell numbers are typically proportional to the viral load (see below), the latter infection rate subsumes cell-cell transmission [29, 30]. A fraction *f*_*D*_ of these infections results in non-productively infected cells, *D*, which do not produce virions. Over 95% of these cells are estimated to be abortively infected and quickly die due to pyroptosis [31, 32].The remaining are long-lived latently infected cells with defective or intact but silent proviruses. In an untreated infection, the contribution to viremia from the reactivation of the latent reservoir is small. For simplicity, we therefore did not consider the latent reservoir separately and neglected the potential reinfection of non-productively infected cells. The remaining fraction, 1−*f*_*D*_, results in productively infected cells, *I*, which produce virions at the rate *pI*. The productively infected cells die due to virus-induced cytopathicity at the rate *d*_*I*_*I* or due to killing by virus-specific CD8 T-cells, *E*, at the rate *kEI*. Free virions are cleared at the rate *d*_*V*_*V*. The cells *E* are produced at the rate *λ*_*E*_ and die at the rate *d*_*E*_*E*. They proliferate with the rate constant *α*_*E*_ and display a saturating dependence on the antigen level for activation, with *θ*_*E*_ the half-maximal saturation constant. The killing rate constant, *k*, depends on the quality of the effector response. For a given effector population *E*, a more focused effector response would imply a higher *k*. *k* can thus vary with time due to clonal expansion, memory recall, exhaustion, and/or viral evolution [14, 33–35]. Immune escape and exhaustion may cause *k* to decline. With time, the rate of escape slows down as the breadth of the immune response increases [36–41]. On the other hand, the ability to recognize new viral epitopes in chronic infection not recognized in primary infection [36, 37, 42–44] can increase *k* with time. Here, we developed an empirical equation to capture these expected patterns of the evolution of *k*. Accordingly, *k* evolves exponentially from an initial value, *k*_*i*_, and saturates at *k*_*f*_, with the changes occurring over the timescale 1⁄*ω*. We tested the various patterns (see below) and found that an increasing *k* starting from *k*_*i*_ = 0 yielded the best fits. CD8 T-cells can also have non-cytolytic effects on infected cells [45, 46]. We considered those effects too (see below), but found that the model above explained the data best (S1 Table).

*S* is the suppressive capacity measured using the *ex vivo* assay. In the experiments, it is estimated as the difference between the antigen load in the CD4 T-cell cultures exposed to the virus in the absence, $\hat{V}(0,\tau )$, and presence, $\hat{V}(\sigma ,\tau )$, of CD8 T-cells, measured at the time *τ*_max_ when the antigen load peaks in the former culture [19]. At any time *t* during the *in vivo* infection, *S* is estimated based on the CD4 and CD8 T-cells drawn from the infected macaque at the time *t* for the *ex vivo* assays. *S* is determined to be a function of *σ*, the elimination rate of infected cells in culture due to CD8 T-cells. *σ* is thus the product of the killing rate constant *k* and the population of CD8 T-cells employed in the assay that are virus-specific. ${\hat{C}}_{0}$ is the total population of CD8 T-cells in the assay, of which the fraction *E/C*_0_ is virus-specific, where *C*_0_ is the total CD8 T-cell concentration in the host. *σ* thus links the *ex vivo* observations with the *in vivo* dynamics. We assumed *k* to be the same *ex vivo* and *in vivo*. Where it has differed in the two settings, factors like prolonged TCR stimulation using viral peptides, isolation of CD8 T-cell clones, and unphysiological effector-to-target cell ratios have been implicated [47–49]. The suppressive capacity assay uses unstimulated CD8 T-cells, does not choose specific clones, and measures their responses to autologous CD4 T-cells instead of viral peptides, rendering it a close mimic of the scenario *in vivo* [19] and justifying our assumption. The other parameters in the expressions for $\hat{V}(\sigma ,\tau )$ and *τ*_max_ are associated with the *ex vivo* assay (S7 Table) and are described in the Methods along with a detailed derivation of the expressions for *S*, $\hat{V}(\sigma ,\tau )$(Methods) and *τ*_max_ (S2 Text).

The above model offered the unified framework necessary for the simultaneous analysis of longitudinal *in vivo* measures of viral dynamics and *ex vivo* CD8 T-cell suppressive capacity. We applied the model to the analysis of data from SIV-infected macaques.

### Model recapitulated dynamics of all the markers

**Fig 2:**
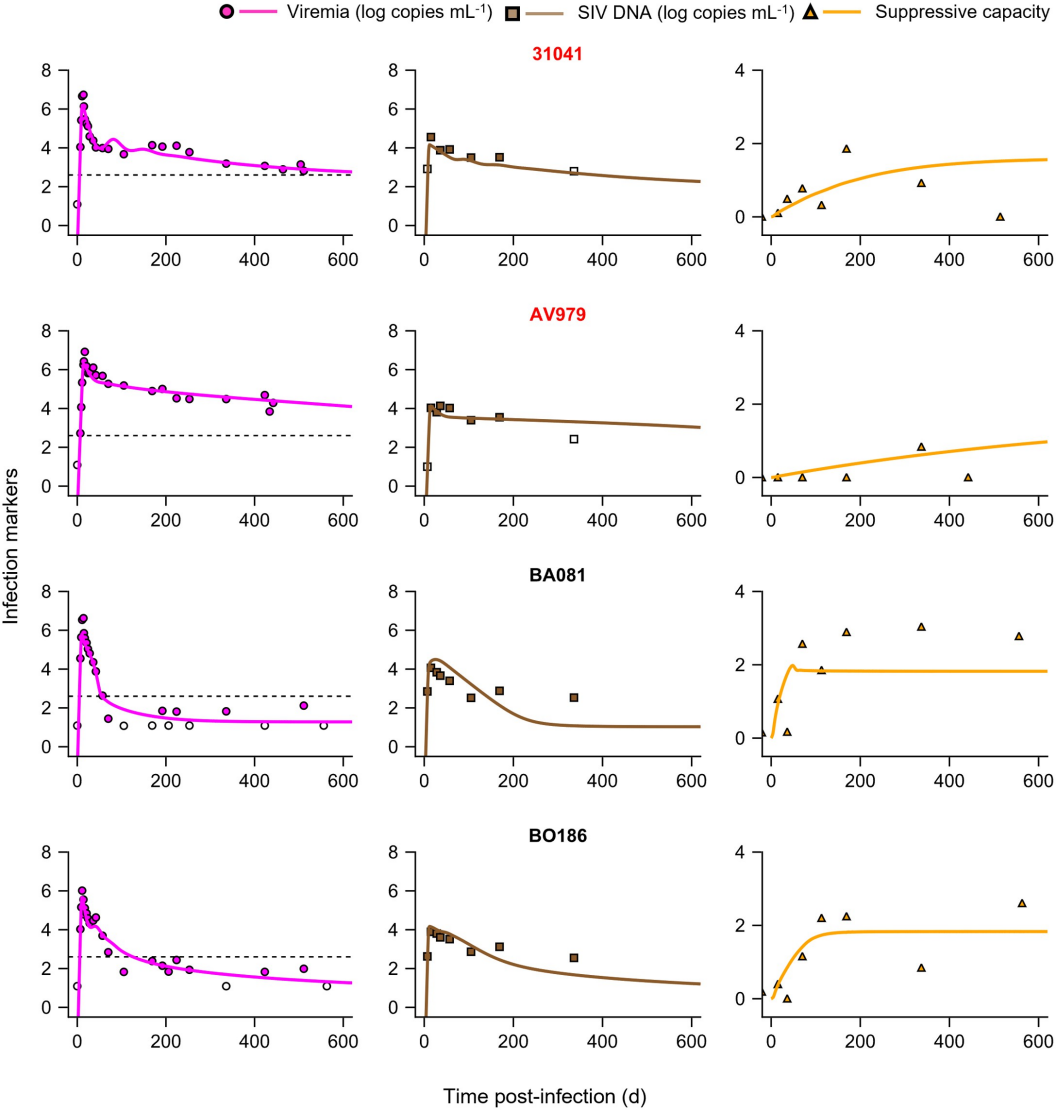
Model fits longitudinal *in vivo* virological and *ex vivo* suppressive capacity data. Model predictions (lines) from simultaneous fitting of the best-fit model (Methods) to all the three datasets (symbols), namely, viremia (left panels), SIV DNA (middle panels) and suppressive capacity (right panels). Macaques highlighted in red were progressors while those in black were controllers. The dashed line in the left panels indicates 400 copies mL^-1^. Open symbols are below the limit of detection. The predictions for the remaining 12 macaques are presented in S10 Fig. The resulting population parameter estimates are in Table 1 and individual parameter estimates are in S10 Table.

We considered longitudinal data of plasma viremia, SIV DNA levels and CD8 T-cell suppressive capacity from 16 cynomolgus macaques infected with SIV (Methods). We fit our model to the data using a nonlinear mixed effects approach (Methods). Our model provided excellent fits to the data (Figs 2 and S10). The estimated population parameters for the best-fit model are in Table 1, and the individual macaque parameters are in S10 Table. The parameter estimates were consistent with previous reports, where available (see Discussion). All the measurements, *in vivo* and *ex vivo*, were thus recapitulated by our model.

Controllers in the experiment were identified as macaques that brought the viral load below 400 copies mL^-1^ after the primary infection phase (~3 months post-exposure) and maintained it below this limit throughout [14] (Methods). By this definition, the dataset had 12 controllers and 4 progressors (or non-controllers). Our model fits yielded set-point viral loads above 400 copies mL^-1^ in the four progressors and below 400 copies mL^-1^ in all controllers, consistent with the experimental observations. Sensitivity analysis showed that these predictions were robust to parameter variations (S11 Fig). We also fit models with three variants of the equation for *k*: constant (*dk/dt =* 0) (S6 Fig and S6 Table), decreasing with time (*k*(0) = *k*_*i*_ > *k*_*f*_ in Eq (6)) (S7 Fig and S7 Table), and initially rising and then falling to a plateau ($k=k_{1}\left(1-e^{-\omega_{1}t}\right)-k_{2}\left(1-e^{-\omega_{2}t}\right)$)(S8 Fig and S8 Table). We found that the increasing form explained the data best (S1 Table). We also considered non-cytolytic effects of CD8 T-cells and found that the present data best supported a model that did not explicitly incorporate them (S1 Text and S9 Fig and S9 Table). Using our best-fit model and parameter estimates, we assessed next the differences between controllers and progressors, focusing on CD8 T-cell responses.

**Table 1:** Population parameter estimates for the best-fit model. Estimates of the parameters from fitting the best-fit model (model #1, S1 Table) to the macaque data (Fig 2). Percent standard errors are in parentheses. *d*_*I*_, *θ*_*E*_, and *d*_*E*_ were fixed based on previous studies (Methods). Random effects for log_10_ *β*’ and log_10_ *T*(0) were removed as they were estimated to be below 0.1 (Methods).

Parameter (Units)	Fixed effect	Random effect
*λ*(cells mL^-1^ d^-1^)	352.70 (35.7)	1.32 (20.1)
log_10_ *β’* (log mL cells^-1^ d^-1^)	-2.84 (0.52)	-
*f*_*D*_	0.93 (0.61)	0.17 (19.7)
${\text{log}}_{10}\lambda_{E}^{*}$(log d^-2^)	0.15 (53)	0.25 (45.5)
*d*_*I*_ (d^-1^)	0.10	-
*d*_*D*_ (d^-1^)	6.9×10^−2^ (27)	0.69 (26.6)
*γ* (cells^-1^)	427.65 (25.7)	0.66 (32.1)
*α*_*E*_ (d^-1^)	0.63 (14.9)	0.20 (71.5)
*θ*_*E*_ (cells mL^-1^)	0.10	-
*d*_*E*_ (d^-1^)	1.00	-
log_10_ *ω*(log d^-1^)	-2.50 (6.38)	0.47 (24.2)
log_10_*T*(0) (log cells mL^-1^)	4.21 (0.39)	-

### CD8 T-cell responses had greater antiviral capacity in controllers than progressors

**Fig 3:**
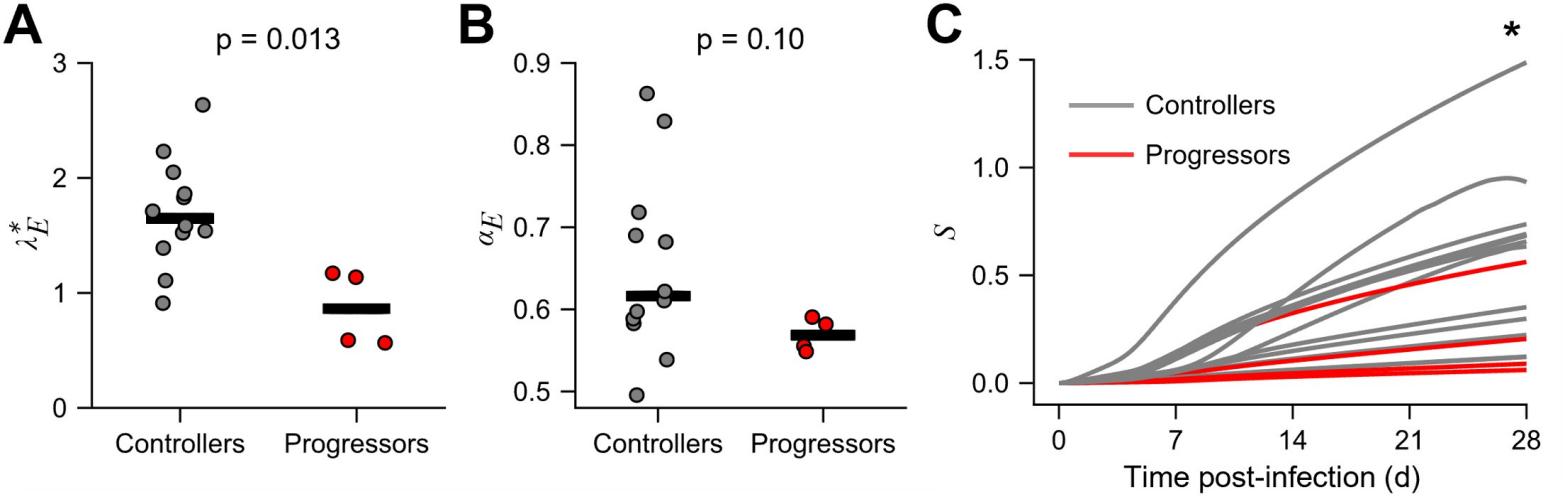
Natural controllers elicit stronger CD8 T-cell responses than progressors. Best-fit model predictions (Fig 2) showed a higher **(A)** recruitment/killing rate and **(B)** antigen-induced proliferation rate of CD8 T-cells in controllers (gray) compared to non-controllers (red). Each symbol represents a macaque and the bar is the median. **(C)** Predictions using the best-fit parameters showed higher suppressive capacity in controllers than non-controllers. * indicates p = 0.04 at the last time point using a Mann-Whitney U test.

Comparing best-fit parameter estimates, we found that controllers had a significantly higher recruitment rate and/or maximal killing rate of CD8 T-cells, contained in the composite parameter $\lambda_{E}^{*}=\lambda_{E}k_{f}$, than progressors (Fig 3A). (We estimated the composite parameter $\lambda_{E}^{*}=\lambda_{E}k_{f}$ because *k*_*f*_ was not independently identifiable; see Methods for details.) Specifically, the median value of $\lambda_{E}^{*}$ was 1.65 d^-2^ in controllers and 0.86 d^-2^ in progressors, implying a nearly 2-fold enhancement in controllers (p = 0.013). Controllers also had a higher antigen-induced proliferation rate of CD8 T-cells (*α*_*E*_), although the latter difference was not significant (Fig 3B). Thus, the CD8 T-cell response seemed more robust in controllers. The controllers, also, interestingly, had a lower value of the ratio of viral production and clearance rates, *γ*(S12 Fig), possibly due to innate immune responses or other cytokine-mediated effects which curtail viral production [14]. The other parameters were not significantly different between the groups (S12 Fig). Here, our aim was to assess whether CD8 T-cell responses would yield a correlate of natural control, notwithstanding other factors. We therefore focused on the differences in $\lambda_{E}^{*}$ and *α*_*E*_, which would manifest as a difference in the suppressive capacity of the CD8 T-cells. Using the best-fit parameter estimates, we predicted the early time-course of the suppressive capacity, *S* for all the macaques and found that the predicted *S* was significantly higher at day 28 in the controllers than progressors (Fig 3C). This suggested that the early suppressive capacity of the CD8 T-cells could be a predictor of natural control. We evaluated this possibility next.

### Cumulative antiviral capacity of the early CD8 T-cell response was correlated with viral control

**Fig 4:**
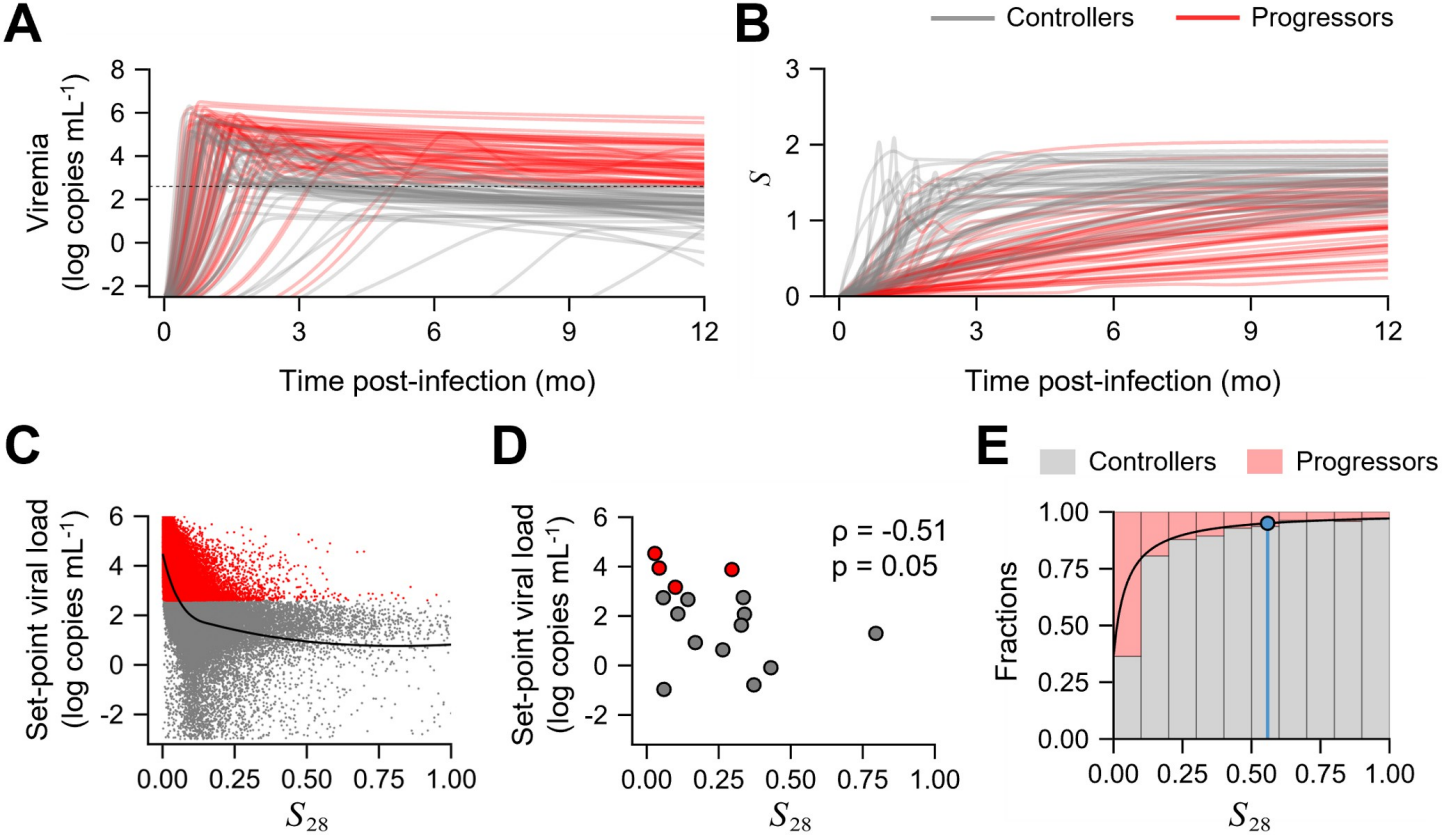
Early cumulative suppressive capacity is a marker of natural control. Dynamics of **(A)** viremia and **(B)** suppressive capacity predicted for virtual patients using our best-fit model. Trajectories for fifty controllers and fifty progressors are shown. Black dashed line indicates 400 copies mL^-1^. Correlation between set-point viral load and cumulative suppressive capacity *S*_28_ (see text) for **(C)** 100000 simulated individuals and **(D)** the 16 macaques studied. The black curve in **(C)** is a LOESS regression curve to visualize the inverse correlation. **(E)** The fraction of virtual individuals achieving control (gray bars) or experiencing progressive disease (red bars) as a function of *S*_28_. Each bar has of width 0.1 units of *S*_28_. The black curve is a fit of the estimated fractions to a first-order Hill function (Methods). The blue line represents the minimum *S*_28_ for >95% controllers, with control defined as set-point viral load <400 copies mL^-1^. Spearman’s ρ was calculated for assessing the correlations.

By sampling parameter values from the distributions obtained in our fits above (Methods), we generated a virtual population of 10^5^ macaques and simulated the progression of SIV infection in each using our model (Fig 4A and 4B). We found that the range of set-point viral loads realized (10^0^–10^6^ copies mL^-1^) was consistent with the range observed in individuals with HIV [50]. For each virtual macaque, we computed the time-averaged area-under-the-curve of *S* over the first 28 days of infection, which we denoted *S*_28_. We found, interestingly, that *S*_28_ was inversely correlated with the set-point viral load (Fig 4C). Thus, early CD8 T-cell responses with greater antiviral capacity were associated with lower set-point viral loads. Using data of the set-point viral loads from the 16 macaques above and the corresponding best-fit predictions of *S*, we found that the above correlation held also in the macaques we studied (Fig 4D). Thus, the cumulative antiviral capacity of the early CD8 T-cell response was a correlate of viremic control.

A model that did not incorporate the suppressive capacity measurements (S1 and S13 Tables) could fit viral load data well (S13 and S14 Figs), as is the case with available models [26, 27, 51], but could not distinguish between the CD8 T-cell responses in controllers and progressors (S15A Fig) and, therefore, could not identify the above correlate (S15B Fig). Since the model was not constrained by any information about the CD8 T-cell function, the per-capita antigen-dependent proliferation rate of CD8 T-cells was estimated to be higher in progressors in response to their higher viral loads (S14 Fig). Moreover, not incorporating the suppressive capacity data to fit the best model resulted in large random effects on parameter estimates (S16 Fig and S13 and S14 Tables). Constraining the model by all the three datasets thus seems to explain a larger proportion of variability in parameter estimates between individuals than when only viral load and SIV DNA data are used. This highlights the importance of our modeling framework, which allows simultaneous fitting of the *ex vivo* suppressive capacity measurements and the *in vivo* viral load and SIV DNA measurements.

The correlate was robust to the duration (28 d) for evaluating the early CD8 T-cell response. For instance, the correlate held when 42 d was used instead of 28 d; the correlation between *S*_42_ and set-point viral load was as strong as the correlation with *S*_28_ (S17A Fig). The correlation was expectedly lost when the time period was too short or long. When the period was too short (14 d, *S*_14_), a significant CD8 T-cell response was yet to be mounted, whereas when it was too long (90 d, *S*_90_), the early dynamics were masked by the dynamics in the chronic phase (S17A Fig).

We asked next whether a threshold *S*_28_ existed that was associated with the set-point viral load of 400 copies mL^-1^ and could thus facilitate distinguishing controllers from progressors as defined in the experiments [14]. We found from the above virtual population that as *S*_28_ increased, the fraction of macaques that exhibited control increased (Fig 4E). The fraction was ~40% when *S*_28_ was <0.1 and rose to ~95% when *S*_28_ was ~0.6. Thus, we defined 0.6 as the critical *S*_28_, above which the chance of achieving viremic control was >95% in our predictions. We recognize that the threshold *S*_28_ would depend on the level of viremia used to define control; a more stringent definition (set-point viremia lower than 400 copies mL^-1^) would lead to a higher threshold (S17B Fig). Nonetheless, once the set-point viremia for control is defined, the corresponding threshold *S*_28_ identified by our model offers a novel, measurable, early predictor of natural control.

## Discussion

Identifying correlates of natural control of HIV infection has been a long-standing goal. Here, combining mathematical modeling and analysis of longitudinal *in vivo* and *ex vivo* data from SIV-infected cynomolgus macaques, we identified the cumulative response of CD8 T-cells during the first 4–6 weeks of infection as an early, measurable marker of natural control. The more efficient was the early CD8 T-cell response, measured in terms of its cumulative virus suppressive capacity, the lower was the set-point viral load. The marker was robust to the duration (~4–6 weeks) over which the early CD8 T-cell response was measured. To our knowledge, this is the first study to identify a quantitative marker predictive of long-term natural control without antiretroviral treatment.

We made significant advances in mathematical modeling that enabled the identification of the marker. The model had to contend with data that was a combination of *in vivo* virological and *ex vivo* immunological measurements. Furthermore, the measurements involved nested time courses. Specifically, each CD8 T-cell suppressive capacity measurement was obtained from time course data of antigen load from *ex vivo* cultures. Longitudinal measurements of suppressive capacity during the *in vivo* infection thus had *ex vivo* assay time course datasets nested within each measurement. Mathematical models thus far have not analyzed such combined *in vivo-ex vivo* datasets. Besides, standard fitting algorithms cannot routinely handle nested time course datasets. By analyzing the *ex vivo* assays, we developed an analytical expression that yielded the suppressive capacity as a function of the killing rate of infected cells by CD8 T-cells. This eliminated the need to consider the *ex vivo* time courses. Recall that the suppressive capacity is obtained as the difference in the antigen level in the *ex vivo* assays in the CD4 T-cell culture and CD4-CD8 T-cell coculture at the time instant when the antigen level peaks in the CD4 culture. Our analytical expression directly predicted this difference, without the need to analyze the entire *ex vivo* time-courses, as a function of the CD8 T-cell killing rate. Consequently, like the plasma viral load, the CD8 T-cell suppressive capacity became a quantity that could be predicted by our model; it was a function of the parameters, specifically the CD8 T-cell killing rate, and other quantities in the model such as the size of the effector pool. Conversely, the suppressive capacity measurements could be used simultaneously with the *in vivo* measurements to fit the model and constrain parameters. Compared to available models [8,26,27,51], which typically rely on viral load and SIV DNA measurements alone, an extra dimension of information by way of the CD8 T-cell suppressive capacity measurements thus became accessible for constraining our model. This mechanism-based learning allowed more accurate inferences of the *in vivo* dynamics and, in particular, enabled identification of the above marker of natural control, which available models missed.

The quality of the fits (Fig 2) as well as the consistency of the best-fit parameter values with independent estimates, where available, gave us further confidence in our inferences. For instance, the best-fit value of the fraction of infection events resulting in non-productively infected cells (*f*_*D*_) was 0.93, close to independent estimates of 95% infection events turning abortive [31,32,51]. The best-fit initial target cell concentration was ~16 cells μL^-1^, which corresponds to ~3% of the baseline CD4 T-cell count in blood (median 654 cells μL^-1^; ref [14]), again consistent with ~5% of the CD4 T-cells in blood expressing CCR5 [52], required for SIV_mac251_ infection. The best-fit ratio of viral production and clearance rates, *γ*, was ~400, consistent with previous reports [26,53,54]. The best-fit timescale of the evolution of the quality of the CD8 T-cell response (ln 2/*ω*) was ~220 days. While the processes driving this timescale are yet to be established, it was comparable to the timescale of evolution of viral diversity (months to years) [33,34].

Our study offers new insights into the potential role of CD8 T-cells in establishing natural control of HIV infection. While several studies have measured CD8 T-cell responses during infection, including in its early stages, the measurements have proven inadequate to distinguish between controllers and progressors [14,18]. Thus, despite the recognition of the importance of CD8 T-cells, a major gap existed in our understanding of their specific role in natural control. Our study makes an important advance by accounting more comprehensively for the antiviral activity of CD8 T-cells than has been done thus far in describing virus dynamics. Our formalism considered not only the quality and the quantity of the CD8 T-cell response but also its time course during the infection. The cumulative antiviral capacity of the CD8 T-cell response early (~4–6 weeks) in infection thus emerged as a correlate of long-term virus control.

In the experiments, although the suppressive capacity was observed to increase in the controllers with time, the rise was significant only at late time points, precluding the identification of the correlate [14]. Specifically, the suppressive capacity was similar between controllers and progressors until 169 days post infection, although most of the controllers suppressed viremia within 90 days. The signatures of the differences in the early time points between the controllers and progressors were elucidated by the model fits. Future studies that may make more frequent measurements in the early phase of the infection may offer a more rigorous experimental test of our correlate.

In our analysis, the higher antiviral capacities in controllers were attributable to greater recruitment rates and/or maximal killing rates of CD8 T-cells compared to progressors. Future studies may also assess further the specific implications of this early cumulative response, such as the restriction of the latent reservoir [55,56], the prevention or reversal of CD8 T cell exhaustion [25,26,55], and/or the formation of an adequate memory pool [14], that may underlie the long-term control realized.

We anticipate implications of our findings for the ongoing efforts to elicit long-term HIV remission [4]. First, using the distribution of parameter values based on fits of our model to the macaque data, our study identified a threshold strength of the marker (*S*_28_) for achieving a set-point viremia representative of long-term control. Future studies may translate this threshold to humans, thereby predicting quantitative targets for interventions aimed at eliciting potent early CD8 T-cell responses for achieving lasting control of HIV-1 infection. Such interventions include vaccination strategies [17] as well as immunotherapies with immune checkpoint inhibitors [57] and broadly-neutralizing antibodies [58] aimed at eliciting better CD8 T-cell responses. Second, early interventions with ART have been shown to increase the chances of achieving post-treatment control [35,59]. While CD8 T-cell responses have been implicated in the establishment of such control [25,26,35,59], how ART may trigger such responses is unclear. Our study suggests that supplementing measurements of viral load with those of *ex vivo* CD8 T-cell suppressive capacity may help elucidate the underlying mechanisms. Such data could be analyzed using the modeling framework developed in our study. The analysis may help understand whether natural control and post-treatment control are the same state realized via two different routes or are two fundamentally distinct states. If the correlation between CD8 T-cell responses early after treatment cessation and the ensuing set-point viral load were similar to that observed in the present study, then post-treatment control would likely be the same state as natural control. Then the notion of the threshold CD8 T-cell response we identified here may be translated also to the post-treatment control scenario. This would further inform the many strategies being explored today that combine ART with other interventions, the latter often designed to improve CD8 T-cell responses, to achieve long-term control [4,17,60].

Our study has limitations. First, although our model is complex, it considered the most parsimonious description of *in vivo* dynamics based on the data available. It thus did not include processes like CD8 T-cell exhaustion or memory. While the model successfully recapitulated the datasets from the untreated macaques that we examined, extending it to treated macaques may require explicitly considering the latter processes. Such advances may also alter the dynamical features of the model—for instance, by introducing bistability [25,26,61]–the implications of which remain to be ascertained. Second, we employed an empirical framework to describe the evolution of the quality of the CD8 T-cell response with time. Again, while such a framework may be adequate for recapitulating natural control, a mechanistic framework, involving phenomena such as CD8 T-cell clonal expansion, differentiation to memory phenotypes and recall [14,62], may be required in other scenarios. Third, the data used in this study was from a non-human primate cohort that had an unrealistically large percentage of natural controllers compared to what is observed in humans [7,63]. In our virtual populations, we ascertained that the range of set-point viral loads predicted by our model was consistent with humans. Yet, to translate the threshold value of the cumulative suppressive capacity to humans, parameters recapitulating not only the range but also the distribution of set-point viremia in humans would have to be employed. Fourth, the present data did not support a model that explicitly considered non-cytolytic effects of CD8 T-cells. This was consistent with observations of the loss of suppressive capacity when contact between CD8 T-cells and infected cells was eliminated in the *ex vivo* assays [9,14]. Yet, the role of non-cytolytic effects of CD8 T-cells, which have been observed *in vivo* [45,46,64,65], cannot be ruled out. Given that non-cytolytic effects appear to be predominant with infected cells that are yet to become productive, deduced through modeling *in vivo* datasets [46], future studies that enable segregation of the infected cells into their pre-productive and productive subsets, both *in vivo* and *ex vivo*, may help refine our model and delineate more accurately the relative contributions of the cytolytic and non-cytolytic effects of CD8 T-cells. Fifth, the *ex vivo* suppressive capacity measurements were made with stock SIV_mac251_ virus [14]. Virus evolution is thus not accounted for in these measurements. Future studies may employ autologous viruses for measuring suppressive capacity. Our model could still be applied to such data, with best-fit estimates of the CD8 T-cell killing rate accounting for the effects of such evolution.

In summary, our study identified a new, robust early marker of natural control of HIV infection, which not only advances our understanding of the mechanisms driving such control but also informs ongoing efforts to devise strategies for eliciting lasting HIV remission.

## Methods

### Model development

#### *Model of* ex vivo *virus dynamics*

We first considered the *ex vivo* assay of the CD8 T-cell suppressive capacity measurements. Here, a fixed number of target CD4 T-cells drawn from an individual is exposed to free virions in culture either in the absence or the presence of a fixed number of autologous CD8 T-cells drawn simultaneously and the time course of the antigen load in the supernatant is measured. We developed a model to predict the latter time course. The suppressive capacity is estimated as the extent to which the antigen load is reduced in the presence of CD8 T-cells compared to its peak level in their absence.

The following equations describe the virus dynamics in a culture of target CD4 T-cells exposed to free virions (Fig 1A):

$$\begin{array}{l} \frac{d\hat{T}}{d\tau }=-\hat{\beta }\hat{T}\hat{V}\\ \frac{d{\hat{I}}_{e}}{d\tau }=(1-f)\hat{\beta }\hat{T}\hat{V}-\rho {\hat{I}}_{e}\\ \frac{d{\hat{I}}_{v}}{d\tau }=\rho {\hat{I}}_{e}-\delta {\hat{I}}_{v}\\ \frac{d\hat{V}}{d\tau }=\hat{p}{\hat{I}}_{v}-d\hat{V} \end{array}$$ (11)

Here, target cells, $\hat{T}$, get infected by virus $\hat{V}$ at the rate $\hat{\beta }\hat{T}\hat{V}$. A fraction 1 − *f* of these infection events is productive, giving rise to infected cells in the eclipse phase, ${\hat{I}}_{e}$, from which virus production is yet to occur. The remaining fraction of the infection events results in non-productively infected cells. These latter cells are assumed to be rendered non-susceptible because they comprise abortively infected cells, which die due to pyroptosis, as well as cells that undergo CD4 downregulation [66,67]. Infected cells in the eclipse phase transition to virus-producing cells, ${\hat{I}}_{v}$, at the rate $\rho {\hat{I}}_{e}$, and produce free virions at the rate $\hat{p}{\hat{I}}_{v}$. Free virions are cleared at the rate $c\hat{V}$. The elimination rate of virus-producing cells is $\delta {\hat{I}}_{v}$. *τ* is the time from the start of the infection in culture. Further, we use the viral load, $\hat{V}$, as a proxy for the antigen load, such as p24 (capsid protein) or p27 (non-structural regulatory protein) levels used as a marker of viral production [19] in the assay.

Because virus production and clearance are fast compared to infection [29, 30], we assumed a quasi-steady state for the dynamics of $\hat{V}$ in Eq (11), so that $\phi =\hat{p}\rho /c$ and $\hat{V}=\hat{p}{\hat{I}}_{v}/c$. In the experimental study, *ex vivo* time courses were measured only in a few cases in order to estimate the time point at which the antigen level would peak in the CD4 T-cell culture [14]. We fit the *ex vivo* model to data from these cases. The model fit the data well (S1A Fig), recapitulating the rise and fall of antigen with time, and yielded estimates of *ρ* (S11 Table). In all the other cases, suppressive capacity was estimated by sampling the *ex vivo* cultures only at the fixed time point at which the antigen level was expected to peak.

Next, to estimate the suppressive capacity, we applied the same model as above to data from co-cultures with a 1:1 mixture of CD4 T-cells and CD8 T-cells exposed to the virus. We let the elimination rate of virus-producing cells in Eq (11) be $(\sigma +\delta ){\hat{I}}_{v}$, where *δ* is the death rate constant of infected cells due to virus-induced cytopathicity and *σ* is the increase in the death rate constant due to CD8 T-cells. Because the population of CD8 T-cells or their killing efficiency is not expected to change during the timeframe of the assays, *σ* can be assumed to be a constant. Our model fit the few available instances of the time-evolution of the antigen load in the co-culture assays as well (S1B Fig), yielding estimates of *σ*. From the fits, the difference in the antigen load at the time when the antigen level peaks in the CD4 T-cell monoculture can be calculated, linking *σ* to the reported suppressive capacity, *S*, of the CD8 T-cells.

The above procedure, however, estimates *σ* by analyzing the entire time-course of the monoculture and co-culture assays at any *in vivo* measurement time point (Fig 1), which would render data fitting challenging. We therefore developed approximations that would yield an analytical expression linking *σ* and *S*. The approximations would also enable more robust analysis of longitudinal datasets of *S* obtained by making single measurements in each *ex vivo* assay at the assay time point associated with the peak antigen load (see above).

#### *Linking* ex vivo *suppressive capacity to* in vivo *killing rate of CD8 T-cells*

Our approach was the following. First, we derived an analytical expression of the time-evolution of the antigen load in the *ex vivo* assay. Second, we obtained an analytical expression of the time at which the antigen load would attain its peak in the monoculture, i.e. with *σ* = 0, denoted *τ*_max_. With these two expressions, we predicted the difference in the antigen load between the monoculture and co-culture assays at the time when the load peaked in the monoculture, thus yielding *S* as a function of *σ*. We present details below.

We recognized that the target cell population remained close to its initial value nearly all the way until the peak in the infection in the monoculture (S1A Fig). We therefore assumed that the target cell population was constant, i.e., $\hat{T}(\tau )={\hat{T}}_{0}$, the initial target cell concentration, until the peak. This transformed our nonlinear model equations in (11) into the set of linear equations below:

$$\begin{array}{l} \frac{d{\hat{I}}_{v}}{d\tau }=(1-f)\hat{\beta }{\hat{T}}_{0}\hat{V}-\rho {\hat{I}}_{v}\\ \frac{d\hat{V}}{d\tau }=\phi {\hat{I}}_{v}-(\sigma +\delta )\hat{V} \end{array}$$ (12)

Solving equations in (12) for the viral load yielded

$$\hat{V}(\sigma ,\tau )=\frac{{\hat{V}}_{0}e^{-\tau (\sigma +\delta +\rho )/2}}{2\alpha }\left((\sigma +\delta -\rho +\alpha )e^{-\tau \alpha /2}+(\rho -\sigma -\delta +\alpha )e^{\tau \alpha /2}\right)$$ (13)

where $\alpha =\sqrt{{\left(\sigma +\delta -\rho \right)}^{2}+4(1-f)\hat{\beta }{\hat{T}}_{0}\phi }$. Predictions with this approximation (Eq (13)) agreed well with the true solution (Eq (11)) of the antigen load until the peak (S1C Fig).

Next, we recognized, following epidemiological models [68,69], that the peak in the infection occurs when the effective reproductive ratio equals 1. Using next-generation matrix methods [68, 69], we derived an analytical expression for the effective reproductive ratio (S2 Text). This yielded *τ*_max_ as the time when the reduction in the target cell population due to the infection would drive the effective reproductive ratio to 1 (S2 Text):

$$\tau_{\text{max}}=\frac{2}{\alpha_{n}-\rho }\text{ln}\left[\frac{\alpha_{n}}{{\hat{V}}_{0}\hat{\beta }}\frac{\rho +\alpha_{n}}{\rho -\alpha_{n}}\text{ln}\left(\frac{\rho \delta }{(1-f)\hat{\beta }\phi {\hat{T}}_{0}}\right)\right]$$ (14)

where $\alpha_{n}=\sqrt{\rho^{2}+4(1-f)\hat{\beta }{\hat{T}}_{0}\phi }$. Combining the expressions of $\hat{V}$ and *τ*_max_ yielded the desired link between *S* and *σ*:

$$S(\sigma )={\text{log}}_{10}\left(\hat{V}(0,\tau_{\text{max}})\right)-{\text{log}}_{10}\left(\hat{V}(\sigma ,\tau_{\text{max}})\right)$$ (15)

where $\hat{V}$ follows from Eq (13).

Estimates of *S*(*σ*) obtained from Eq (15) were close to those obtained by integrating Eq (11) (S1D Fig).

#### *Model building strategy for* in vivo *dynamics*

To identify the number of SIV DNA compartments our within-host models should contain, we fit mono-, bi-, and tri-exponential curves to the post-peak SIV DNA data. The SIV DNA data we used did not differentiate between unintegrated and integrated (intact and defective) SIV DNA. If *z*(*t*) represents the DNA level at time *t*, then a multi-exponential function is given by

$$z(t)=\sum_{i=1}^{m}z_{i}e^{-\nu_{i}t}$$ (16)

Here, *m* is the number of phases (or compartments), *ν*_*i*_ represents the decay rate constant of the *i*^th^ phase, and *z*_*i*_ is the constant pre-factor for the *i*^th^ phase, respectively. The initial condition $z(0)=\sum_{i=1}^{m}z_{i}$ is the estimated DNA level in the blood at *t* = 0, the time point where the measurement peaked for the macaque. We found that a bi-exponential curve explained the data best (S18 Fig). Moreover, accounting for only a single SIV DNA compartment yielded poorer fits (S19 Fig and S15 Table). Accordingly, we incorporated two SIV DNA compartments in our *in vivo* models.

Next, we constructed several models to describe the *in vivo* dynamics with two SIV DNA compartments (S1 Text). We compared these models by fitting data. The best model, with the lowest Bayesian Information Criterion (BIC), is described in the Results. We analyzed the model for its structural identifiability and applied it to fit data.

### Structural identifiability of model parameters and data fitting

Before fitting our models to data, we analyzed their identifiability in the following way. For each model, we first examined the structural identifiability using the differential-algebraic elimination method implemented in the Julia package StructuralIdentifiability.jl [70]. Structurally non-identifiable parameters are fixed to values obtained from the literature. Structural identifiability does not guarantee practical identifiability, the latter dependent also on the datasets available. To ensure practical identifiability, we iterated the inference process in Monolix to identify the subset of the remaining free parameters that were responsible for practical non-identifiability. Fixing them, again using literature values, resulted in full identifiability. We thus had to fix the parameters *d*_*I*_, *θ*_*E*_, and *d*_*E*_ to make the remaining parameters of our best model uniquely identifiable. Additional parameters had to be fixed in other tested models (S1 Table), as they involved more parameters (S2–S10, S12, S13, and S15 Tables).

From previous studies, we fixed *θ*_*E*_ = 0.1 cells mL^-1^ and *d*_*E*_ = 0.1 d^-1^ [25,71]. We fixed *d*_*I*_ = 0.1 d^-1^ based on recent estimates of the half-life of productively infected cells (1.0 d to 1.7 d) [51,65,72] and estimates of >40% of infected cell loss attributable to CD8 T-cell function [73]. Note that our model prediction of the set-point viral load was not sensitive to *d*_*I*_ (S11 Fig).

In the *in vivo* model, *k*_*f*_ was not identifiable. So, we applied the transformation E* = *k*_*f*_*E*. Also, viral production and clearance happen at a much faster rate than other *in vivo* processes [29,30]. So, assuming quasi-steady state between virion production and clearance rates [29,30], we simplified the equation for viremia, giving us *pI ≈ d*_*V*_*V ⇒ V(t) = γI(t)* where *γ* = *p/d*_*V*_. These transformations to the *in vivo* model combined with the analytical expression linking *S* and *σ* for the *ex vivo* measurements yielded

$$\frac{dT}{dt}=\lambda -\beta \prime TI-d_{T}T$$ (17)

$$\frac{dI}{dt}=(1-f_{D})\beta \prime TI-K^{*}E^{*}I-d_{I}I$$ (18)

$$\frac{dD}{dt}=f_{D}\beta \prime TI-d_{D}D$$ (19)

$$\frac{dE^{*}}{dt}=\lambda_{E}^{*}+\alpha_{E}E^{*}\frac{I}{\theta_{E}+I}-d_{E}E^{*}$$ (20)

$$\frac{dK^{*}}{dt}=\omega (1-K^{*})$$ (21)

$$S(\sigma )={\text{log}}_{10}\left(\hat{V}\left(0,\tau_{\text{max}}\right)\right)-{\text{log}}_{10}\left(\hat{V}\left(\sigma ,\tau_{\text{max}}\right)\right)$$ (22)

$$\hat{V}(\sigma ,\tau )=\frac{{\hat{V}}_{0}e^{-\frac{\tau (\sigma +\delta +\rho )}{2}}}{2\alpha }\left(\left(\sigma +\delta -\rho +\alpha \right)e^{-\frac{\tau \alpha }{2}}+\left(\rho -\sigma -\delta +\alpha \right)e^{\frac{\tau \alpha }{2}}\right)$$ (23)

$$\tau_{\text{max}}=\frac{2}{\alpha_{n}-\rho }\text{ln}\left[\frac{\alpha_{n}}{{\hat{V}}_{0}\hat{\beta }}\frac{\rho +\alpha_{n}}{\rho -\alpha_{n}}\text{ln}\left(\frac{\rho \delta }{(1-f)\hat{\beta }\phi {\hat{T}}_{0}}\right)\right]$$ (24)

$$\sigma =K^{*}{\hat{C}}_{0}\left(\frac{E^{*}}{C_{0}}\right)$$ (25)

where $\alpha =\sqrt{{\left(\sigma +\delta -\rho \right)}^{2}+4(1-f)\hat{\beta }{\hat{T}}_{0}\phi }$ and $\alpha_{n}=\sqrt{\rho^{2}+4(1-f)\hat{\beta }{\hat{T}}_{0}\phi }$. Here, *β*’ = *γβ*, K* = *k*/*k_f_*, E* = *k_f_E* and $\lambda_{E}^{*}=\lambda_{E}k_{f}$. The above Eqs (17–25) were used for data fitting. Parameters used for the *ex vivo* model are presented in S11 Table, and the initial conditions for the *in vivo* model are provided in S16 Table. We note that *d*_*T*_ is fixed by the pre-infection steady state of the uninfected target cells, *T*(0) = *λ*/*d*_*T*_.

### Statistical model for longitudinal data fitting

We employed the nonlinear mixed effects modeling (NLME) approach for fitting longitudinal data and used the implementation of stochastic approximation of expectation-maximization (SAEM) algorithm in Monolix 2021R1 (https://lixoft.com/). Initial conditions for the *in vivo* models are provided in S16 Table. The variables *V* = *γI*, *I* + *D* and *S* were fit to the viremia, SIV DNA, and suppressive capacity datasets, respectively. We assumed random effects for all parameters and removed them if they were less than 0.1. The statistical model describing these observations is

$$\begin{array}{l} y_{ij}^{1}\sim {\text{log}}_{10}\gamma_{i}I_{ij}+a_{1}\varepsilon_{ij}^{1}\\ y_{ij}^{2}\sim {\text{log}}_{10}\left(I_{ij}+D_{ij}\right)+a_{2}\varepsilon_{ij}^{2}\\ y_{ij}^{3}\sim \left(S(\sigma_{ij})\right)+\left(a_{3}+b_{3}S(\sigma_{ij})\right)\varepsilon_{ij}^{3} \end{array}$$ (26)

Here, *y*_*ij*_ represents the observations for the *i*^th^ individual at the *j*^th^ time point. The superscripts 1, 2 and 3 represent the log-transformed viremia, log-transformed SIV DNA and suppressive capacity measurements, respectively. *ε* is the residual Gaussian error with a constant standard deviation. Thus, for viremia and total SIV DNA datasets, we used a constant error model, while for the suppressive capacity data, both constant and proportional error terms were considered. Fits to the best-fit model are presented in Figs 2 and S10, while for the other models, they are presented in S2–S10, S13, S16, and S19 Figs.

### Sensitivity analysis

We performed sensitivity analysis of the set-point viral load estimates of our best-fit model. Sobol’s method was employed using the GlobalSensitivity.jl [74] package in Julia.

### Virtual population

All parameters except for *f*_*D*_, which followed a logit-normal distribution with bounds between 0 and 1, were assumed to follow a log-normal distribution. Consequently, log_10_ *β’*, ${\text{log}}_{10}\;\lambda_{E}^{*}$, log_10_ *ω* and log_10_ *T*(0) followed a normal distribution. After model fitting, analytical forms of the corresponding distributions of the population parameters were used to generate the virtual population (Fig 4A–4E).

The fraction of controllers estimated by our model, plotted in Fig 4E, was fit to a first-order Hill function of *S*_28_ given by $a_{1}+(1-a_{1})\frac{S_{28}}{a_{2}+S_{28}}$ using the nonlinear Levenberg-Marquardt algorithm in Julia. Here, *a*_1_ and *a*_2_ were fit parameters. Accordingly, *a*_1_ is the probability of achieving control in the limit of a negligible early CD8 T-cell response (*S*_28_ → 0) and *a*_2_ is the half-maximal saturation constant.

### Data

We obtained data from a published study [14]. In the study, 16 macaques, of which 6 carried the protective M6 MHC haplotype, were infected with SIV_mac251_ intrarectally. They were then followed for 18 months without any intervention. Throughout this time, viremia, SIV DNA in blood and suppressive capacity of CD8 T-cells were measured at different time points. By the end of the study, 12 of the 16 macaques were identified as controllers. Viremia measurements were made as copies of SIV RNA mL^-1^ of blood. SIV DNA levels per million cells were converted from copies per 10^6^ leukocytes to copies mL^-1^ of blood, using individual blood leukocyte counts sampled simultaneously to the SIV DNA measurements.

## Supporting information

S1 Text *In vivo* model variants. (DOCX)

S2 Text Derivation of *τ*_max_. (DOCX)

S1 FigThe *ex vivo* model predictions and fits.**(A)** Fits (lines) of the *ex vivo* model (Eq (11), main text) to antigen load data (symbols) from CD4 T-cell cultures of 18 samples. Sample IDs are presented on the top of the corresponding panels. Antigen p27 level is assumed to be $\mu \hat{V}$, where $\hat{V}$ is the viral load and *μ* is the amount of antigen per copy of virion. *μ* and *ρ* were identifiable and were estimated to be 6.2×10^−7^ ng copies^-1^ and 0.36 d^-1^, respectively. The pink curves plot the corresponding target cell concentrations. **(B)** Fits of the *ex vivo* model to the 1:1 CD4 and CD8 T-cell co-cultures of 18 samples. Sample IDs are presented on the top of corresponding panels. Estimated *ρ* from fits to CD4 T-cell cultures were used and *σ* was adjusted to fit the model. **(C)** Estimates of viral load in the cultures by Eq (13) from main text (purple) and numerical integration of system in Eq (11) from main text (gray). The CD4 T-cell culture corresponds to *σ* = 0, while the other cases are co-cultures. **(D)** Estimates of the suppressive capacity calculated from the Eq (15) from main text (purple) and the numerical integration of system (Eq (11), main text) (gray).(TIF)

S2 Fig Fits of model #2 to data. Model predictions (lines) from simultaneous fitting of model #2 (Methods; S1 Table) to all the three datasets (symbols), namely, viremia (magenta), SIV DNA (brown) and suppressive capacity (yellow). Macaques highlighted in red are progressors while the rest are controllers. Empty symbols are observations below the limit of detection. The parameter estimates resulting in these fits are in S2 Table.
(TIF)

S3 Fig Fits of model #3 to data. Model predictions (lines) from simultaneous fitting of model #3 (Methods; S1 Table) to all the three datasets (symbols), namely, viremia (magenta), SIV DNA (brown) and suppressive capacity (yellow). Macaques highlighted in red are progressors while the rest are controllers. Empty symbols are observations below the limit of detection. The parameter estimates resulting in these fits are in S3 Table.
(TIF)

S4 Fig Fits of model #4 to data. Model predictions (lines) from simultaneous fitting of model #4 (Methods; S1 Table) to all the three datasets (symbols), namely, viremia (magenta), SIV DNA (brown) and suppressive capacity (yellow). Macaques highlighted in red are progressors while the rest are controllers. Empty symbols are observations below the limit of detection. The parameter estimates resulting in these fits are in S4 Table.
(TIF)

S5 Fig Fits of model #5 to data. Model predictions (lines) from simultaneous fitting of model #5 (Methods; S1 Table) to all the three datasets (symbols), namely, viremia (magenta), SIV DNA (brown) and suppressive capacity (yellow). Macaques highlighted in red are progressors while the rest are controllers. Empty symbols are observations below the limit of detection. The parameter estimates resulting in these fits are in S5 Table.
(TIF)

S6 Fig Fits of the model #6 to data. Model predictions (lines) from simultaneous fitting of model #6 (Methods; S1 Table) to all the two virological datasets (symbols), namely, viremia (magenta) and SIV DNA (brown). Macaques highlighted in red are progressors while the rest are controllers. Empty symbols are observations below the limit of detection. The parameter estimates resulting in these fits are in S6 Table.
(TIF)

S7 Fig Fits of the model #7 to data. Model predictions (lines) from simultaneous fitting of model #7 (Methods; S1 Table) to all the two virological datasets (symbols), namely, viremia (magenta) and SIV DNA (brown). Macaques highlighted in red are progressors while the rest are controllers. Empty symbols are observations below the limit of detection. The parameter estimates resulting in these fits are in S7 Table.
(TIF)

S8 Fig Fits of model #8 to data. Model predictions (lines) from simultaneous fitting of model #8 (Methods; S1 Table) to all the three datasets (symbols), namely, viremia (magenta), SIV DNA (brown) and suppressive capacity (yellow). Macaques highlighted in red are progressors while the rest are controllers. Empty symbols are observations below the limit of detection. The parameter estimates resulting in these fits are in S8 Table.
(TIF)

S9 Fig Fits of model #9 to data. Model predictions (lines) from simultaneous fitting of model #9 (Methods; S1 Table) to all the three datasets (symbols), namely, viremia (magenta), SIV DNA (brown) and suppressive capacity (yellow). Macaques highlighted in red are progressors while the rest are controllers. Empty symbols are observations below the limit of detection. The parameter estimates resulting in these fits are in S9 Table.
(TIF)

S10 Fig Fits of the best-fit model to data. Model predictions (lines) from simultaneous fitting of the best-fit model (Methods; S1 Table) to all the three datasets (symbols), namely, viremia (magenta), SIV DNA (brown) and suppressive capacity (yellow), shown for 12 of 16 macaques. Plots for the remaining 4 macaques are presented in Fig 2. Macaques highlighted in red are progressors while the rest are controllers. Empty symbols are observations below the limit of detection. The parameter estimates resulting in these fits are detailed in Table 1 of the main text and S10 Table.
(TIF)

S11 Fig Sensitivity analysis. Sensitivity of the set-point viral load predicted by the best-fit model to its parameters estimated using Sobol’s method.
(TIF)

S12 Fig Comparison of parameters estimated by model #1. Parameters estimated for all the individuals are grouped based on their control status—controllers vs. progressors—and compared. Presented here are five parameters (*λ*, *d*_*D*_, *α*_*E*_, *f*_*D*_ and log_10_*ω*). The others are in Fig 3. Mann-Whitney U test was used to estimate the significance levels.
(TIF)

S13 Fig Fits of the model that does not incorporate suppressive capacity measurements and constant *k* (model #10) to data. Model predictions (lines) from simultaneous fitting of model #10 (Methods; S1 Table) to all the two virological datasets (symbols), namely, viremia (magenta) and SIV DNA (brown). Macaques highlighted in red are progressors while the rest are controllers. Empty symbols are observations below the limit of detection. The parameter estimates resulting in these fits are in S12 Table.
(TIF)

S14 Fig Comparison of parameters estimated by the model that does not incorporate suppressive capacity measurements (model #10) for fitting. Parameters estimated for all the individuals are grouped based on their control status—controllers vs. progressors—and compared. Mann-Whitney U test was used to estimate the significance levels.
(TIF)

S15 Fig Comparison of CD8 T-cell killing rate between model with and without suppressive capacity. **(A)** Effector response dynamics of CD8 T-cells, given by K*E*, predicted for the macaques by the best-fit model (solid) and model #10, which does not incorporate suppressive capacity measurements for fitting (dashed). **(B)** Correlation plot between *S*_28_ and set-point viral load as predicted by model #10. Gray symbols are controllers, while red symbols are progressors. Spearman’s ρ was calculated for assessing the correlation. Note that here the set-point viral load increases with *S*_28_, which is the opposite of what is expected.
(TIF)

S16 Fig Fits of the main model without incorporating the suppressive capacity measurements (model #11) to data. Model predictions (lines) from simultaneous fitting of model #11 (Methods; S1 Table) to all the two virological datasets (symbols), namely, viremia (magenta) and SIV DNA (brown). Macaques highlighted in red are progressors while the rest are controllers. Empty symbols are observations below the limit of detection. The parameter estimates resulting in these fits are in S13 Table.
(TIF)

S17 Fig Robustness of correlate. **(A) Sensitivity to duration for evaluating the early CD8 T-cell responses.** Correlation between set-point viral load and AUC of suppressive capacity averaged over 14, 42 and 90 days post infection, respectively, for the 16 macaques. Gray symbols are controllers, while red symbols are progressors. The bar plot at the bottom right presents the predicted correlation between set-point viral load and the time-averaged area-under-the-curve of *S* estimated for different durations. Asterisks represent significant correlations with p<0.05; ns: not significant. **(B) Minimum *S***_**28**_ **required for control increases with a stricter definition of control.** The minimum *S*_28_ estimated to be required for 95% likelihood of control as a function of the threshold viral load for control. Spearman’s ρ was calculated for assessing the correlations.
(TIF)

S18 Fig Identifying number of phases of SIV DNA. Mono- (dashed), bi- (solid), and tri-exponential (dotted) curves are fitted to longitudinal SIV DNA data post the peak in the measurements. Empty symbols are below detection limit. Data were fit in Monolix (Methods; main text). The bi-exponential curve explained the data best (BICs: 164.48 for the mono-exponential curve; 140.36 for the bi-exponential curve; and 171.13 for the tri-exponential curve).
(TIF)

S19 Fig Fits of the model with constant *k* and no *D* compartment (model #12) to data. Model predictions (lines) from simultaneous fitting of model #12 (Methods; S1 Table) to all the two virological datasets (symbols), namely, viremia (magenta) and SIV DNA (brown). Macaques highlighted in red are progressors while the rest are controllers. Empty symbols are observations below the limit of detection. The parameter estimates resulting in these fits are in S15 Table.
(TIF)

S1 Table Comparison of different models fitted to the data. Every model fit to the data is summarized, comparing the BICs of fits. *These fits do not include suppressive capacity datasets and hence cannot be compared with other models directly. S14 Table presents the comparison of BIC of these models with that of the main model after eliminating the contribution from the suppressive capacity data for the latter.
(DOCX)

S2 Table Population parameter estimates for model #2. The fixed and random effects of each parameter is provided along with respective percent standard errors in parentheses. In addition to the parameters fixed in model #1, *f*_*D*_ is fixed to 0.95 and *θ*_*X*_ is fixed to 5 cells mL^-1^ [1, 2].
(DOCX)

S3 Table Population parameter estimates for model #3. The Hill coefficient for the exhaustion rate, *n* = 1. The fixed and random effects of each parameter is provided along with respective percent standard errors in parentheses. In addition to the parameters fixed in model #1, *f*_*D*_ is fixed to 0.95, *ϕ* is fixed to 2 and *κ* is fixed to 1 d^-1^ [1–3].
(DOCX)

S4 Table Population parameter estimates for model #4. The Hill coefficient for the exhaustion rate, *n* = 4. The fixed and random effects of each parameter is provided along with respective percent standard errors in parentheses. In addition to the parameters fixed in model #1, *f*_*D*_ is fixed to 0.95, *ϕ* is fixed to 2 and *κ* is fixed to 1 d^-1^ [1–3].
(DOCX)

S5 Table Population parameter estimates for model #5. The fixed and random effects of each parameter is provided along with respective percent standard errors in parentheses. In addition to the parameters fixed in model #1, *f*_*D*_ is fixed to 0.95 [1].
(DOCX)

S6 Table Population parameter estimates for model #6. The fixed and random effects of each parameter is provided along with respective percent standard errors in parentheses. Similar to the best-fit model (Table 1), parameters *d*_*I*_, *θ*_*E*_ and *d*_*E*_ were fixed.
(DOCX)

S7 Table Population parameter estimates for model #7. The fixed and random effects of each parameter is provided along with respective percent standard errors in parentheses. Similar to the best-fit model (Table 1), parameters *d*_*I*_, *θ*_*E*_ and *d*_*E*_ were fixed. In addition, log_10_ *ω* was fixed to -2.50 from Table 1.
(DOCX)

S8 Table Population parameter estimates for model #8. The fixed and random effects of each parameter is provided along with respective percent standard errors in parentheses. Similar to the best-fit model (Table 1), parameters *d*_*I*_, *θ*_*E*_ and *d*_*E*_ were fixed. In addition, log_10_ *β’* and log_10_ *T*(0) were fixed using values from Table 1.
(DOCX)

S9 Table Population parameter estimates for model #9. The fixed and random effects of each parameter is provided along with respective percent standard errors in parentheses. In addition to the parameters fixed in model #1, *f*_*D*_ is fixed to 0.95 [1].
(DOCX)

S10 Table Individual parameter estimates for the best-fit model. Fixed parameters are *d*_*I*_, *θ*_*E*_ and *d*_*E*_ respectively [1,2], as detailed in Methods of main text. Random effects for log_10_ *β*’ and log_10_*T*(0) were less than 0.1, and were thus removed, rendering them to be same across macaques.
(DOCX)

S11 Table Parameters of the *ex vivo* model. The table lists the values used, and the references thereof. CD8 T-cell count in untreated SIV-infected cynomolgus macaques was close to 10^6^ cells mL^-1^ [5], similar to the levels in HIV-infected humans [6,7]. So, we fixed *C*_0_ to 10^6^.
(DOCX)

S12 Table Population parameter estimates for model #10. The fixed and random effects of each parameter are provided along with respective percent standard errors in parentheses. In addition to the parameters fixed in the best-fit model, *f*_*D*_ is fixed to 0.95 [1].
(DOCX)

S13 Table Population parameter estimates for model #11. The fixed and random effects of each parameter are provided along with respective percent standard errors in parentheses. In addition to the parameters fixed in the best-fit model, *f*_*D*_ is fixed to 0.95 [1].
(DOCX)

S14 Table Comparison of model fits without suppressive capacity data. Contribution of suppressive capacity data to BIC of model #1 was removed to compare it with models #10 and #11.
(DOCX)

S15 Table Population parameter estimates for model #12. The fixed and random effects of each parameter is provided along with respective percent standard errors in parentheses.
(DOCX)

S16 Table Initial conditions used for *in vivo* model fitting. Note that the exhaustion compartment, *Q*, is present only in models #3 and #4. Viral inoculum sizes have been estimated using the volumes of distribution [1].
(DOCX)
