# Supplementary material for: Antiviral capacity of the early CD8 T-cell response is predictive of natural control of SIV infection: Learning in vivo dynamics using ex vivo data
Source: PLoS Comput Biol. 2024 Sep 10;20(9):e1012434. doi: 10.1371/journal.pcbi.1012434 (PMC11414924; doi:10.1371/journal.pcbi.1012434)
Supplement: S1 Text — (DOCX) [file pcbi.1012434.s001.docx]

# TEXT S1. *IN VIVO* MODEL VARIANTS

We describe our model (Model #1) that best describes the data (summarized from the main text), followed by its variants. The following equations constitute Model #1:

where and .

The differential equations in (S1) encode the *in vivo* portion of the framework. Briefly, these equations describe the following processes. The level of uninfected target cells is at homeostatic equilibrium in the absence of infection, which is altered upon exposure to the virus. Target cells are either productively or non-productively infected by free virions. CD8 T-cell responses control the infection by killing productively infected cells. The per-capita *in vivo* killing rate of CD8 T-cells is modeled to rise exponentially with time post-exposure until saturation. The algebraic equations in (S1) describe the suppressive capacity assay and link the *ex vivo* assay measurements to the *in vivo* killing rate of CD8 T-cells. Assuming quasi-steady state between virus production and clearance rates yields , where . Also, based on identifiability analysis (Methods; main text), variables and are scaled as and . These transformations yield the final form of the framework that is fit to the data (equations (17)-(25) of the main text.)

Variants of the *in vivo* portion of the framework were considered based on previous studies on HIV control [1-5]. For simplicity, the variants are presented below without repeating the *ex vivo* equations. First, following Conway and Perelson [1], we built Model #2 (equation (S2)) with SIV-specific effector CD8 T-cells getting exhausted at the rate . The other equations in the model are identical to those in model #1.

$$\begin{matrix} \frac{dT}{dt}=\lambda-\beta TV-d_{T}T \\ \frac{dI}{dt}=\left(1-f_{D}\right)\beta TV-kEI-d_{I}I \\ \frac{dD}{dt}=f_{D}\beta TV-d_{D}D \\ \frac{dV}{dt}=pI-d_{V}V \\ \frac{dE^{*}}{dt}=\lambda_{E}^{*}+\alpha_{E}E^{*}\frac{I}{\theta_{E}+I}-\left(\alpha_{E}+\alpha_{R}\right)E^{*}\frac{I}{\theta_{X}+I}-d_{E}E \\ \frac{dK^{*}}{dt}=\omega\left(1-K^{*}\right) \end{matrix}$$

Here, is the exhaustion rate constant, and is the half-maximal saturation constant for the exhaustion rate dependent on the instantaneous antigen level. To ensure that the estimate of is larger than [1, 2], we used and constrained to be strictly positive.

Model #3 tracks the exhaustion level of CD8 T-cells, *Q*, explicitly, following Johnson et al. [3]. is dependent on the cumulative level of antigenic stimulation rather than the instantaneous level, in accordance with experiments [4]. The equations for model #3 are:

Here, is the maximum rate at which the exhaustion level rises, is the rate constant for the reversal of exhaustion, is the maximum rate at which the effector cells suffer exhaustion and is the corresponding half-maximal saturation constant. The Hill coefficient for exhaustion, , is 1 [3]. We note here that cells that reverse exhaustion are implicitly assumed to join the effector pool. Following previous formalisms [1, 3], we do not consider exhausted cells explicitly. Instead, we let the overall level of exhaustion dictate the size of the effector pool. Thus, high levels of exhaustion, determined by the quantity $Q$, would shrink the effector pool, $E$. Conversely, lowering $Q$ would eventually lead to an increase in $E$.

Model #4 is identical to model #3, except for the Hill coefficient, which is set to 4 [5].

In Model #5, following Desikan et al. [5], we explicitly accounted for antigen-dependent enhanced recruitment of effector cells. This changes the equation for CD8 T-cells to , where is the maximum rate of antigen-dependent recruitment. For simplicity, we considered the same half-maximal saturation constant, , for both antigen-dependent recruitment and proliferation events. The other equations in the model are identical to those in Model #3.

Model #6, Model #7, and Model #8 test different functional forms of *k* (constant, decreasing, non-monotonic) described in the main text.

Model #9 explicitly accounts for non-cytolytic effects of CD8 T-cells. Following Cao et al. [6], the non-cytolytic effects are considered to decrease the virus production rate from productively infected cells by a factor $1+\eta E$, where $\eta$ is the strength of the non-cytolytic effects. This yielded

$$\begin{matrix} \frac{dT}{dt}=\lambda-\frac{\beta'}{1+\eta^{*}E^{*}}TI-d_{T}T \\ \frac{dI}{dt}=\left(1-f_{D}\right)\frac{\beta'}{1+\eta^{*}E^{*}}TI-K^{*}E^{*}I-d_{I}I \\ \frac{dD}{dt}=f_{D}\frac{\beta'}{1+\eta^{*}E^{*}}TI-d_{D}D \\ \frac{dE^{*}}{dt}=\lambda_{E}^{*}+\alpha_{E}E^{*}\frac{I}{\theta_{E}+I}-d_{E}E^{*} \\ \frac{dK^{*}}{dt}=\omega\left(1-K^{*}\right) \end{matrix}$$

Here, $\eta^{*}=\eta/k_{f}$ and $V=\frac{\gamma}{1+\eta^{*}E^{*}}I$.

Fits of the above models to the data are in Fig. S2 – S10, and the corresponding parameter estimates are in Table. S2 – S10.

**References**

1. Conway JM, Perelson AS. Post-treatment control of HIV infection. Proc Natl Acad Sci U S A. 2015;112(17):5467-72. Epub 20150413. doi: 10.1073/pnas.1419162112. PubMed PMID: 25870266; PubMed Central PMCID: PMCPMC4418889.

2. Baral S, Antia R, Dixit NM. A dynamical motif comprising the interactions between antigens and CD8 T cells may underlie the outcomes of viral infections. Proc Natl Acad Sci U S A. 2019;116(35):17393-8. Epub 20190814. doi: 10.1073/pnas.1902178116. PubMed PMID: 31413198; PubMed Central PMCID: PMCPMC6717250.

3. Johnson PL, Kochin BF, McAfee MS, Stromnes IM, Regoes RR, Ahmed R, et al. Vaccination alters the balance between protective immunity, exhaustion, escape, and death in chronic infections. J Virol. 2011;85(11):5565-70. Epub 20110316. doi: 10.1128/JVI.00166-11. PubMed PMID: 21411537; PubMed Central PMCID: PMCPMC3094965.

4. Wherry EJ, Kurachi M. Molecular and cellular insights into T cell exhaustion. Nat Rev Immunol. 2015;15(8):486-99. doi: 10.1038/nri3862. PubMed PMID: 26205583; PubMed Central PMCID: PMCPMC4889009.

5. Desikan R, Raja R, Dixit NM. Early exposure to broadly neutralizing antibodies may trigger a dynamical switch from progressive disease to lasting control of SHIV infection. PLoS Comput Biol. 2020;16(8):e1008064. Epub 20200820. doi: 10.1371/journal.pcbi.1008064. PubMed PMID: 32817614; PubMed Central PMCID: PMCPMC7462315.

6. Cao Y, Cartwright EK, Silvestri G, Perelson AS. CD8+ lymphocyte control of SIV infection during antiretroviral therapy. PLoS Pathog. 2018;14(10):e1007350. Epub 20181011. doi: 10.1371/journal.ppat.1007350. PubMed PMID: 30308068; PubMed Central PMCID: PMCPMC6199003.
