# Supplementary material for: Antiviral capacity of the early CD8 T-cell response is predictive of natural control of SIV infection: Learning in vivo dynamics using ex vivo data
Source: PLoS Comput Biol. 2024 Sep 10;20(9):e1012434. doi: 10.1371/journal.pcbi.1012434 (PMC11414924; doi:10.1371/journal.pcbi.1012434)
Supplement: S2 Text — (DOCX) [file pcbi.1012434.s002.docx]

# TEXT S2. DERIVATION OF

In this section, we present the derivation of the time, , at which the antigen load in the CD4 T-cell culture in our *ex vivo* model setup (equation (11), main text) peaks.

Because virus-induced cytopathicity is negligible in these assays [1], we simplified equation (13) in the main text by setting , so that

$$\hat{V}(\tau)\approx\frac{\hat{V}_{0}}{2\alpha_{n}}\left(\left(\rho+\alpha_{n}\right)e^{-\tau(\rho-\alpha_{n})/2}-(\rho-\alpha_{n})e^{-\tau(\rho+\alpha_{n})/2}\right)$$

where . We substituted equation (S1) into the differential equation for , (equation (13), main text), solving which yielded

$$\begin{matrix} \hat{T}(\tau)=\hat{T}_{0}\exp\left(-\frac{4\hat{\beta}\rho\hat{V}_{0}}{\rho^{2}-\alpha^{2}}\right)\times\\ \exp\left(\frac{\hat{V}_{0}\hat{\beta}}{\alpha_{n}}\left[\frac{\rho+\alpha_{n}}{\rho-\alpha_{n}}\exp\left(-\frac{\tau\left(\rho-\alpha_{n}\right)}{2}\right)-\frac{\rho-\alpha_{n}}{\rho+\alpha_{n}}\exp\left(-\frac{\tau\left(\rho+\alpha_{n}\right)}{2}\right)\right]\right) \end{matrix}$$

From Table S11, mL copies^-1^ d^-1^, d^-1^, d^-1^ and copies mL^-1^, which implied that . Further, vanishes quickly compared to . The expression for in equation (S2) therefore simplified to

$$\hat{T}(\tau)\approx\hat{T}_{0}\times\exp\left(\frac{\hat{V}_{0}\hat{\beta}}{\alpha_{n}}\frac{\rho+\alpha_{n}}{\rho-\alpha_{n}}\exp\left(-\tau\left(\rho-\alpha_{n}\right)/2\right)\right)$$

From , the effective reproductive ratio of the virus, , followed as

where is the fraction of the target cells infected by time , and is the basic reproductive ratio, estimated at the start of the infection [2]. is the number of new infected cells that a single infected cell can give rise to in its lifetime [2, 3]. At , the infection spreads at the rate . As increases due to the depletion of target cells, the infection slows down. At the target cell concentration when drops below 1, the infection starts subsiding [2]. Thus, is the time when . In other words, at .

To estimate , we used the next-generation matrix method. From our system of equations for the CD4 T-cell culture (equation (11), main text), we recognized that the infection subsystem (see [3]) will have the following equations for and :

and denote the transmission and transition matrices, respectively, at the infection-free steady state [3], and are defined as

where . denotes the new infection events in the *i*^th^ compartment. , with and the transition events into and out of the *i*^th^ compartment, respectively. The subscript 0 in equation (S6) denotes the evaluation of the partial derivatives at the infection-free steady state ( and ). This yields , , and respectively, and hence

is the spectral radius of the matrix :

Thus,

Combining equations (S3), (S4) and (S9) then yielded

Estimates of , and for the parameter values employed are presented in Table S11.

# References

1. Saez-Cirion A, Shin SY, Versmisse P, Barre-Sinoussi F, Pancino G. Ex vivo T cell-based HIV suppression assay to evaluate HIV-specific CD8+ T-cell responses. Nat Protoc. 2010;5(6):1033-41. Epub 20100513. doi: 10.1038/nprot.2010.73. PubMed PMID: 20539279.

2. Heffernan JM, Smith RJ, Wahl LM. Perspectives on the basic reproductive ratio. J R Soc Interface. 2005;2(4):281-93. doi: 10.1098/rsif.2005.0042. PubMed PMID: 16849186; PubMed Central PMCID: PMCPMC1578275.

3. Diekmann O, Heesterbeek JA, Roberts MG. The construction of next-generation matrices for compartmental epidemic models. J R Soc Interface. 2010;7(47):873-85. Epub 20091105. doi: 10.1098/rsif.2009.0386. PubMed PMID: 19892718; PubMed Central PMCID: PMCPMC2871801.
