# Supplementary figures and images for: Antiviral capacity of the early CD8 T-cell response is predictive of natural control of SIV infection: Learning in vivo dynamics using ex vivo data
Source: PLoS Comput Biol. 2024 Sep 10;20(9):e1012434. doi: 10.1371/journal.pcbi.1012434 (PMC11414924; doi:10.1371/journal.pcbi.1012434)

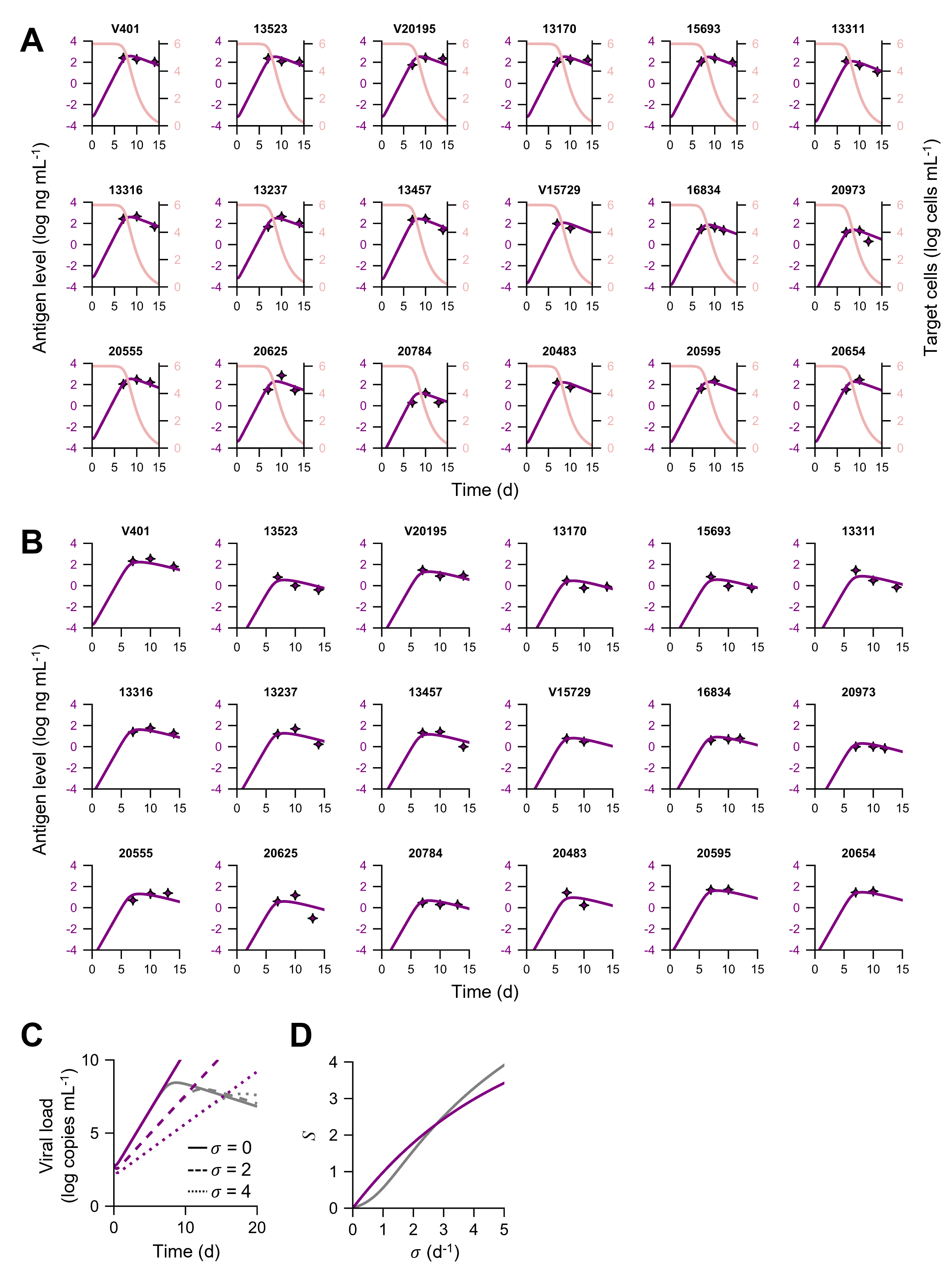

Supplement: S1 Fig — (A) Fits (lines) of the ex vivo model (Eq (11), main text) to antigen load data (symbols) from CD4 T-cell cultures of 18 samples. Sample IDs are presented on the top of the corresponding panels. Antigen p27 level is assumed to be μV^, where V^ is the viral load and μ is the amount of antigen per copy of virion. μ and ρ were identifiable and were estimated to be 6.2×10^−7^ ng copies-1 and 0.36 d-1, respectively. The pink curves plot the corresponding target cell concentrations. (B) Fits of the ex vivo model to the 1:1 CD4 and CD8 T-cell co-cultures of 18 samples. Sample IDs are presented on the top of corresponding panels. Estimated ρ from fits to CD4 T-cell cultures were used and σ was adjusted to fit the model. (C) Estimates of viral load in the cultures by Eq (13) from main text (purple) and numerical integration of system in Eq (11) from main text (gray). The CD4 T-cell culture corresponds to σ = 0, while the other cases are co-cultures. (D) Estimates of the suppressive capacity calculated from the Eq (15) from main text (purple) and the numerical integration of system (Eq (11), main text) (gray). (TIF) [file pcbi.1012434.s003.tif]

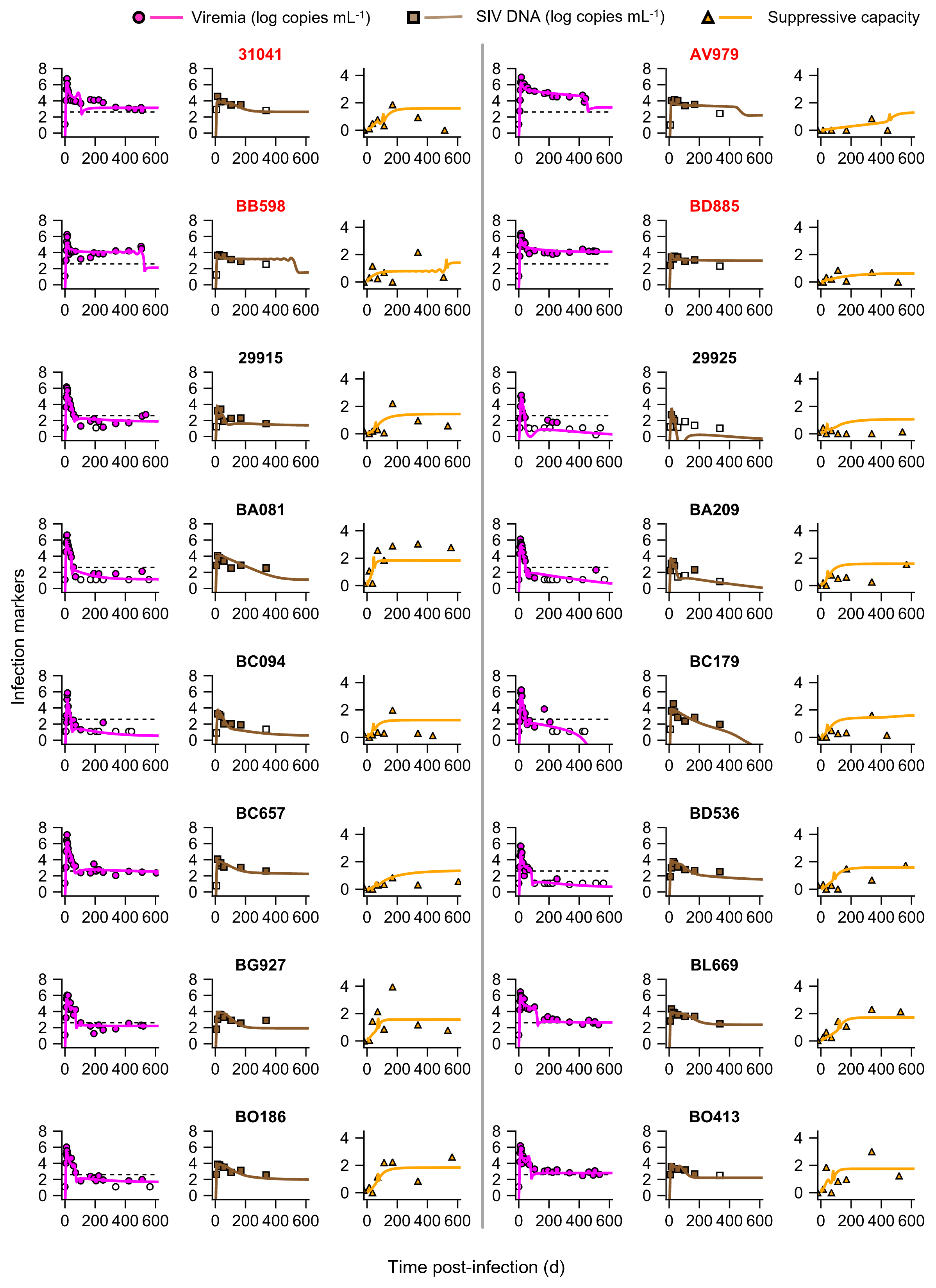

Supplement: S2 Fig — Model predictions (lines) from simultaneous fitting of model #2 (Methods; S1 Table) to all the three datasets (symbols), namely, viremia (magenta), SIV DNA (brown) and suppressive capacity (yellow). Macaques highlighted in red are progressors while the rest are controllers. Empty symbols are observations below the limit of detection. The parameter estimates resulting in these fits are in S2 Table. (TIF) [file pcbi.1012434.s004.tif]

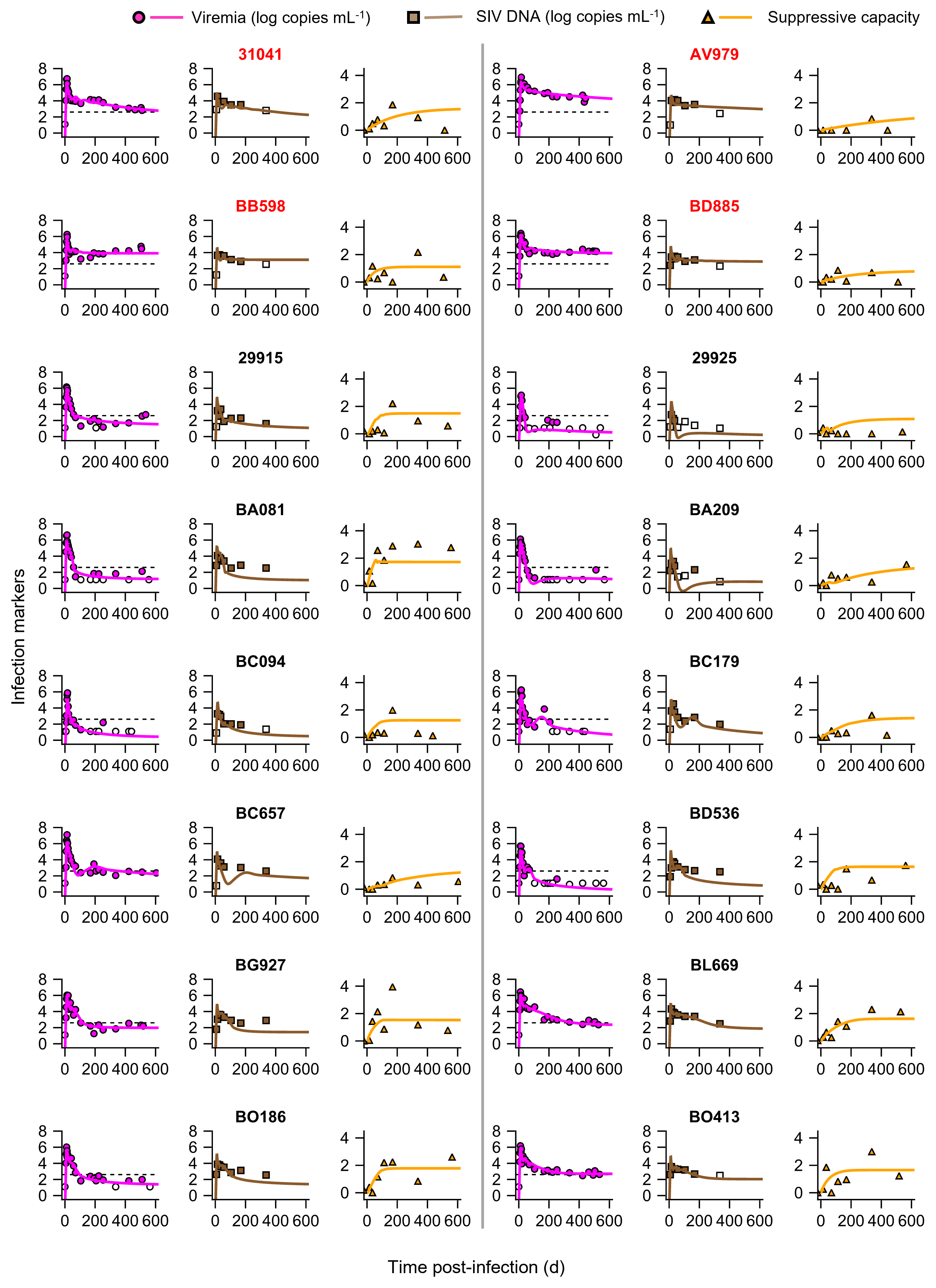

Supplement: S3 Fig — Model predictions (lines) from simultaneous fitting of model #3 (Methods; S1 Table) to all the three datasets (symbols), namely, viremia (magenta), SIV DNA (brown) and suppressive capacity (yellow). Macaques highlighted in red are progressors while the rest are controllers. Empty symbols are observations below the limit of detection. The parameter estimates resulting in these fits are in S3 Table. (TIF) [file pcbi.1012434.s005.tif]

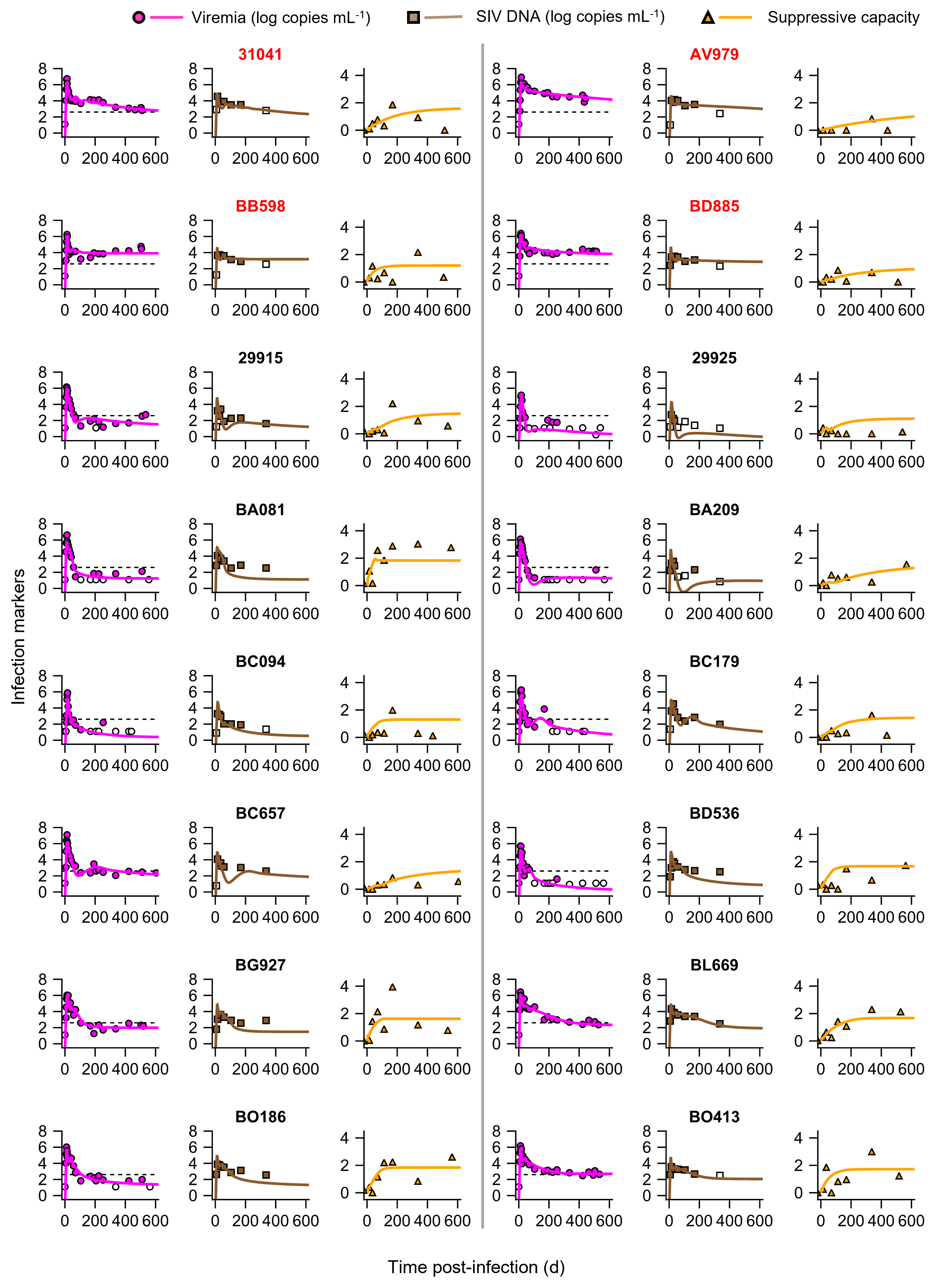

Supplement: S4 Fig — Model predictions (lines) from simultaneous fitting of model #4 (Methods; S1 Table) to all the three datasets (symbols), namely, viremia (magenta), SIV DNA (brown) and suppressive capacity (yellow). Macaques highlighted in red are progressors while the rest are controllers. Empty symbols are observations below the limit of detection. The parameter estimates resulting in these fits are in S4 Table. (TIF) [file pcbi.1012434.s006.tif]

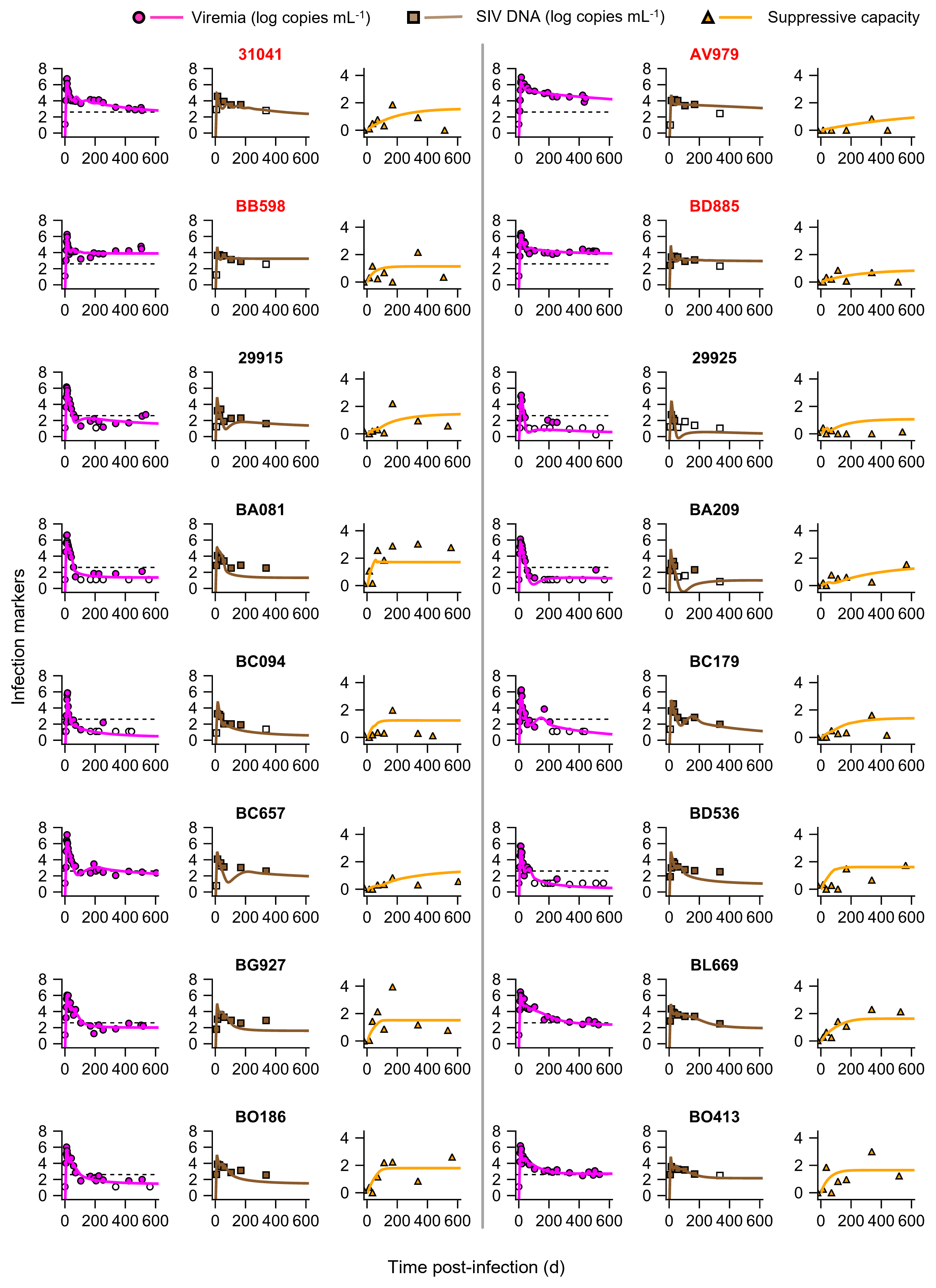

Supplement: S5 Fig — Model predictions (lines) from simultaneous fitting of model #5 (Methods; S1 Table) to all the three datasets (symbols), namely, viremia (magenta), SIV DNA (brown) and suppressive capacity (yellow). Macaques highlighted in red are progressors while the rest are controllers. Empty symbols are observations below the limit of detection. The parameter estimates resulting in these fits are in S5 Table. (TIF) [file pcbi.1012434.s007.tif]

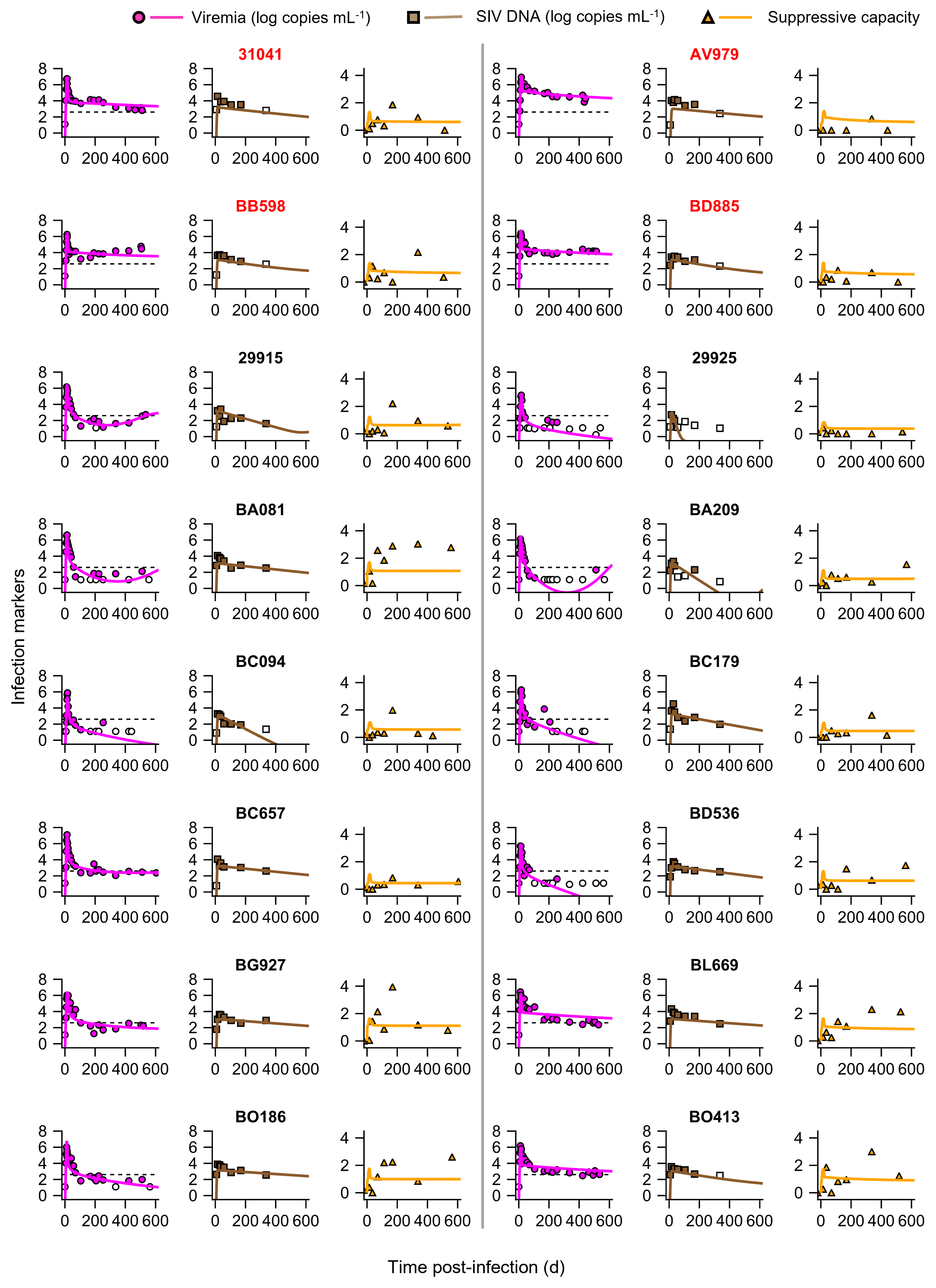

Supplement: S6 Fig — Model predictions (lines) from simultaneous fitting of model #6 (Methods; S1 Table) to all the two virological datasets (symbols), namely, viremia (magenta) and SIV DNA (brown). Macaques highlighted in red are progressors while the rest are controllers. Empty symbols are observations below the limit of detection. The parameter estimates resulting in these fits are in S6 Table. (TIF) [file pcbi.1012434.s008.tif]

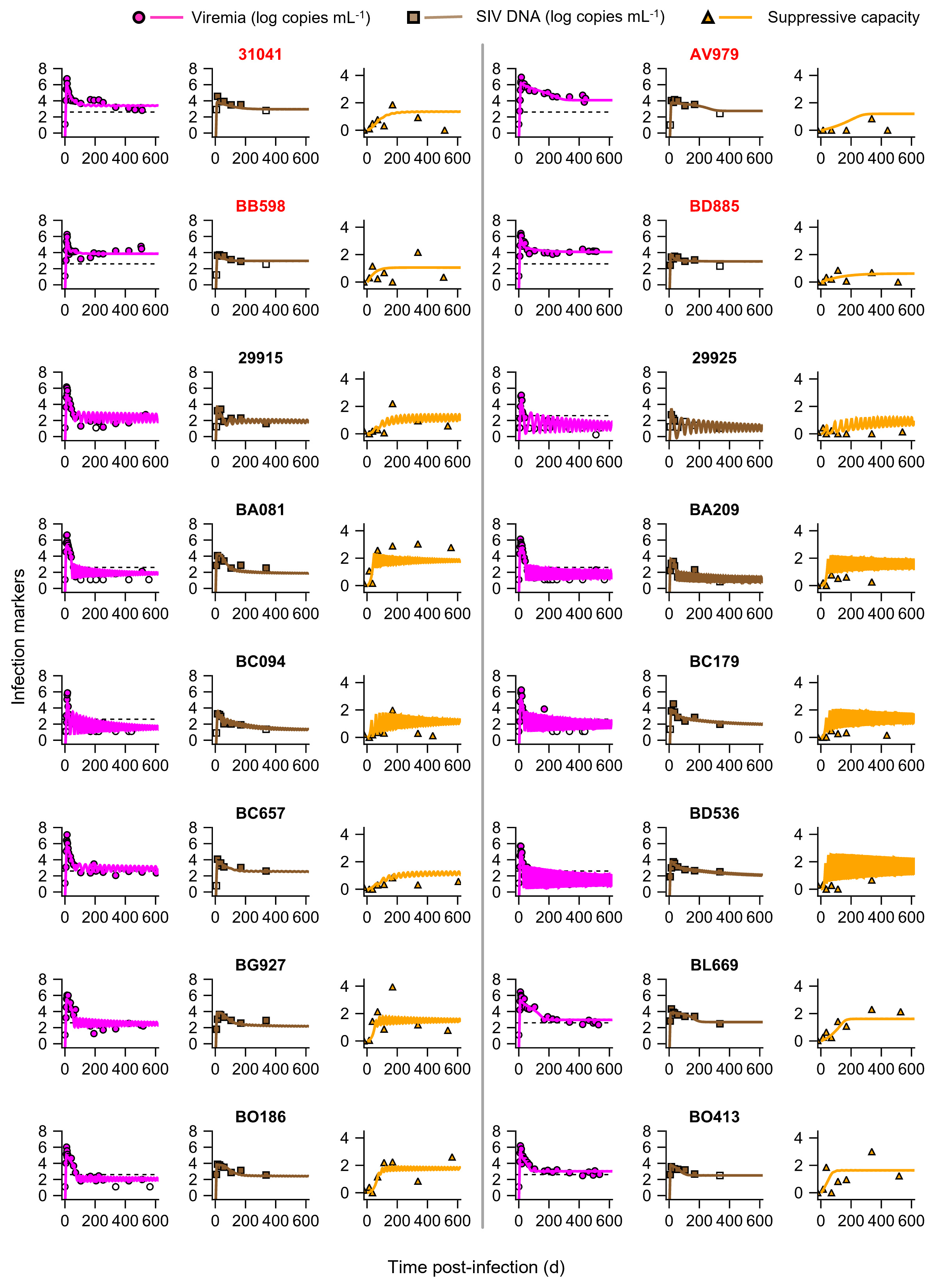

Supplement: S7 Fig — Model predictions (lines) from simultaneous fitting of model #7 (Methods; S1 Table) to all the two virological datasets (symbols), namely, viremia (magenta) and SIV DNA (brown). Macaques highlighted in red are progressors while the rest are controllers. Empty symbols are observations below the limit of detection. The parameter estimates resulting in these fits are in S7 Table. (TIF) [file pcbi.1012434.s009.tif]

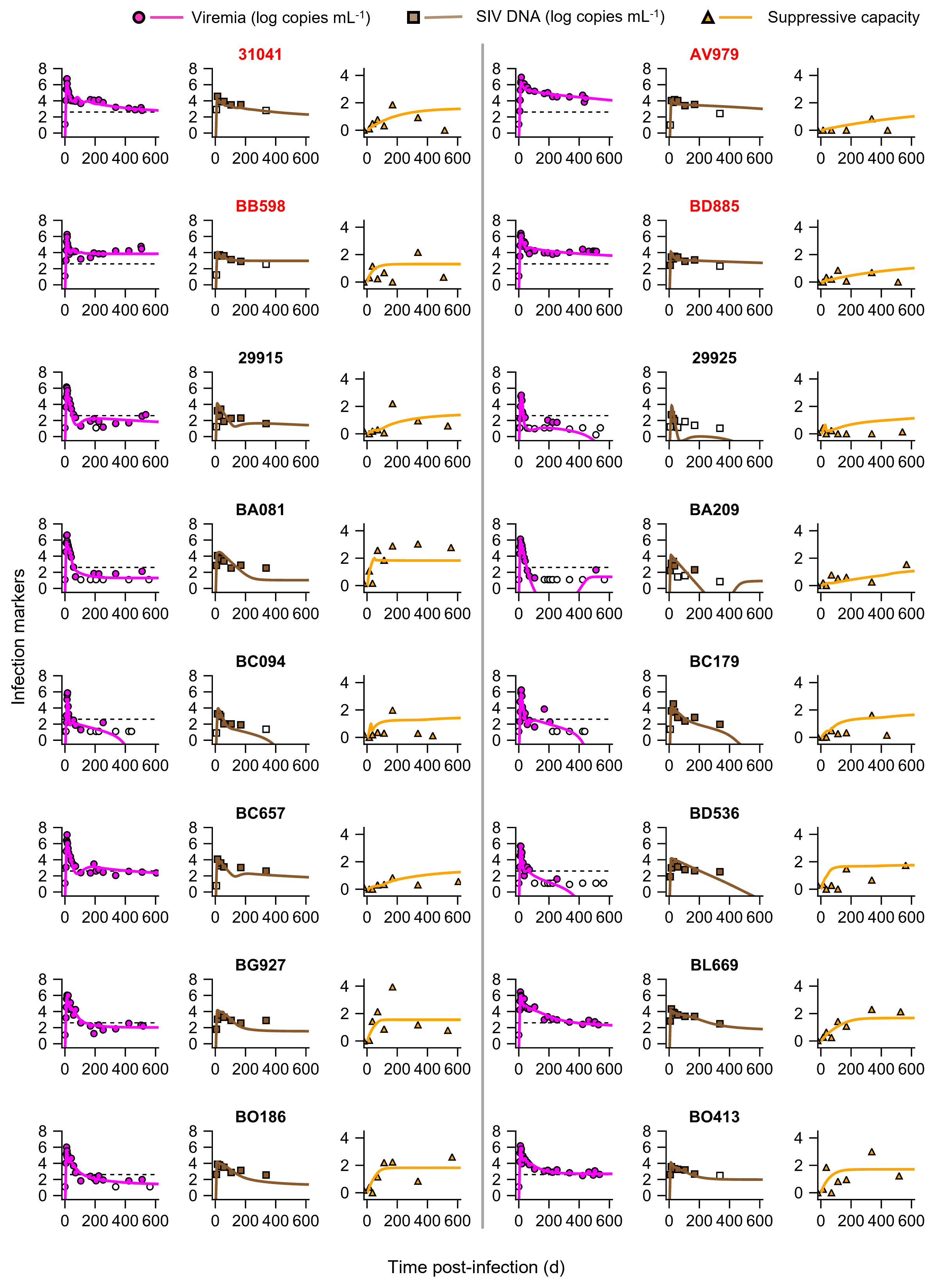

Supplement: S8 Fig — Model predictions (lines) from simultaneous fitting of model #8 (Methods; S1 Table) to all the three datasets (symbols), namely, viremia (magenta), SIV DNA (brown) and suppressive capacity (yellow). Macaques highlighted in red are progressors while the rest are controllers. Empty symbols are observations below the limit of detection. The parameter estimates resulting in these fits are in S8 Table. (TIF) [file pcbi.1012434.s010.tif]

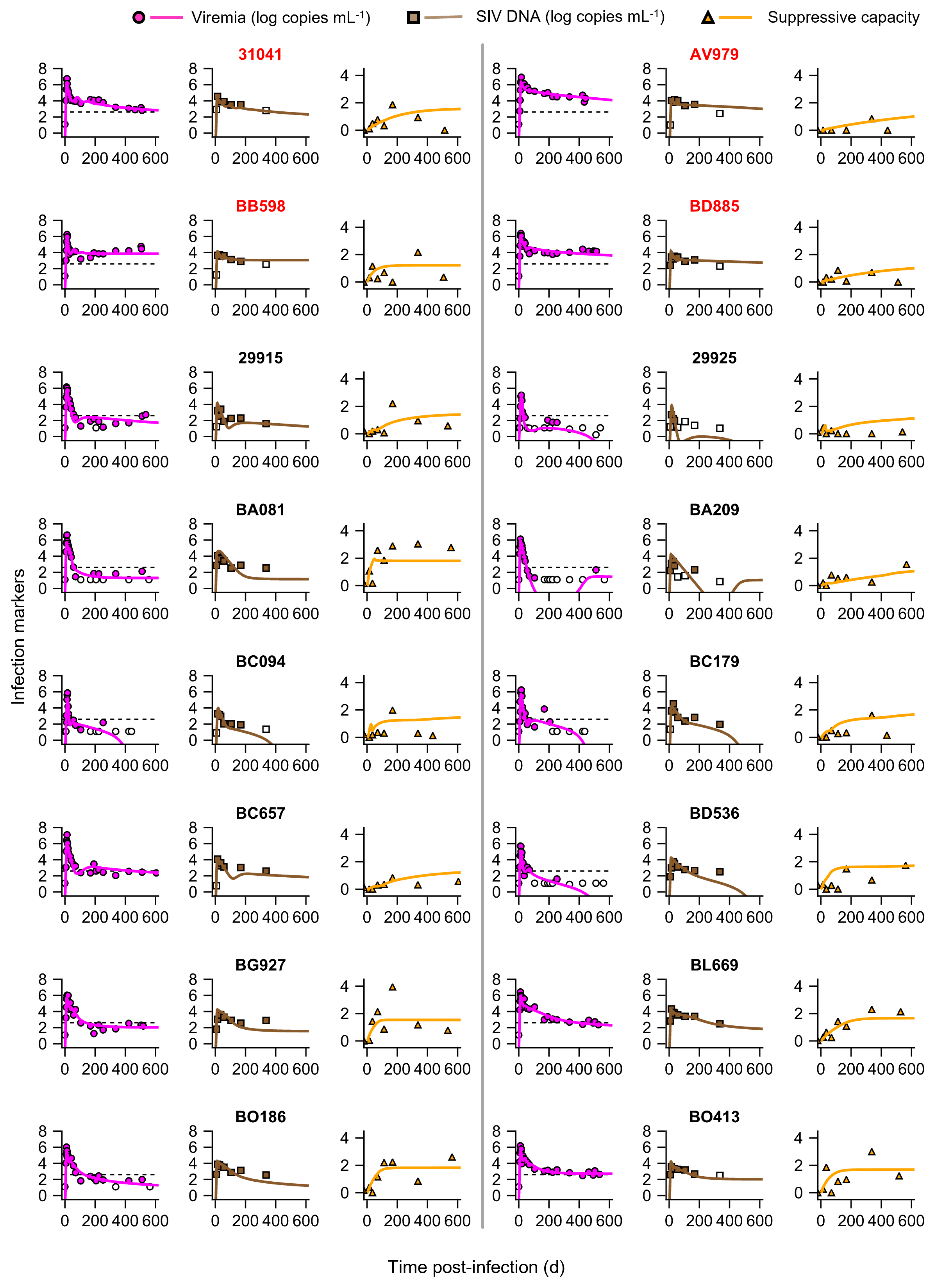

Supplement: S9 Fig — Model predictions (lines) from simultaneous fitting of model #9 (Methods; S1 Table) to all the three datasets (symbols), namely, viremia (magenta), SIV DNA (brown) and suppressive capacity (yellow). Macaques highlighted in red are progressors while the rest are controllers. Empty symbols are observations below the limit of detection. The parameter estimates resulting in these fits are in S9 Table. (TIF) [file pcbi.1012434.s011.tif]

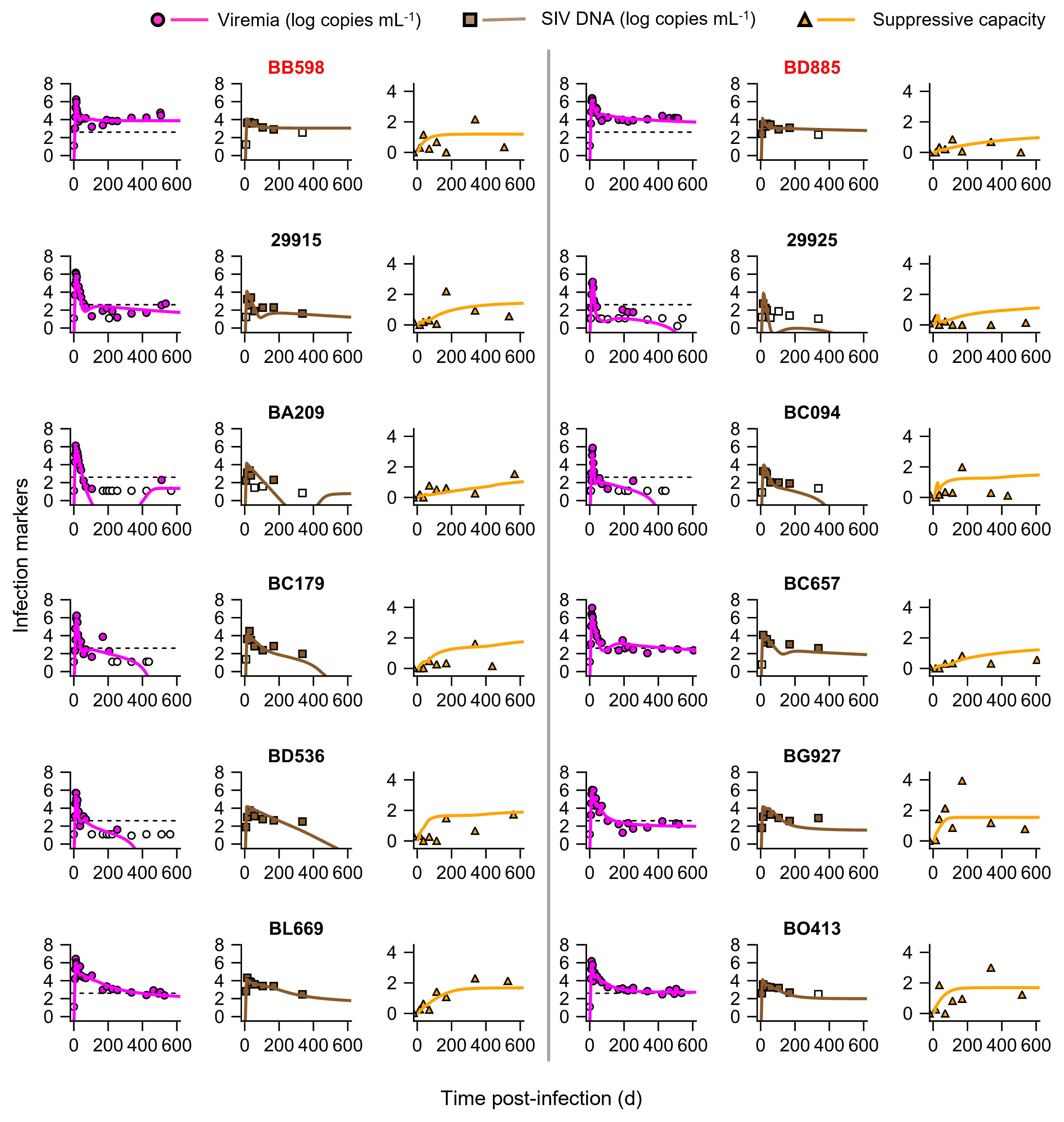

Supplement: S10 Fig — Model predictions (lines) from simultaneous fitting of the best-fit model (Methods; S1 Table) to all the three datasets (symbols), namely, viremia (magenta), SIV DNA (brown) and suppressive capacity (yellow), shown for 12 of 16 macaques. Plots for the remaining 4 macaques are presented in Fig 2. Macaques highlighted in red are progressors while the rest are controllers. Empty symbols are observations below the limit of detection. The parameter estimates resulting in these fits are detailed in Table 1 of the main text and S10 Table. (TIF) [file pcbi.1012434.s012.tif]

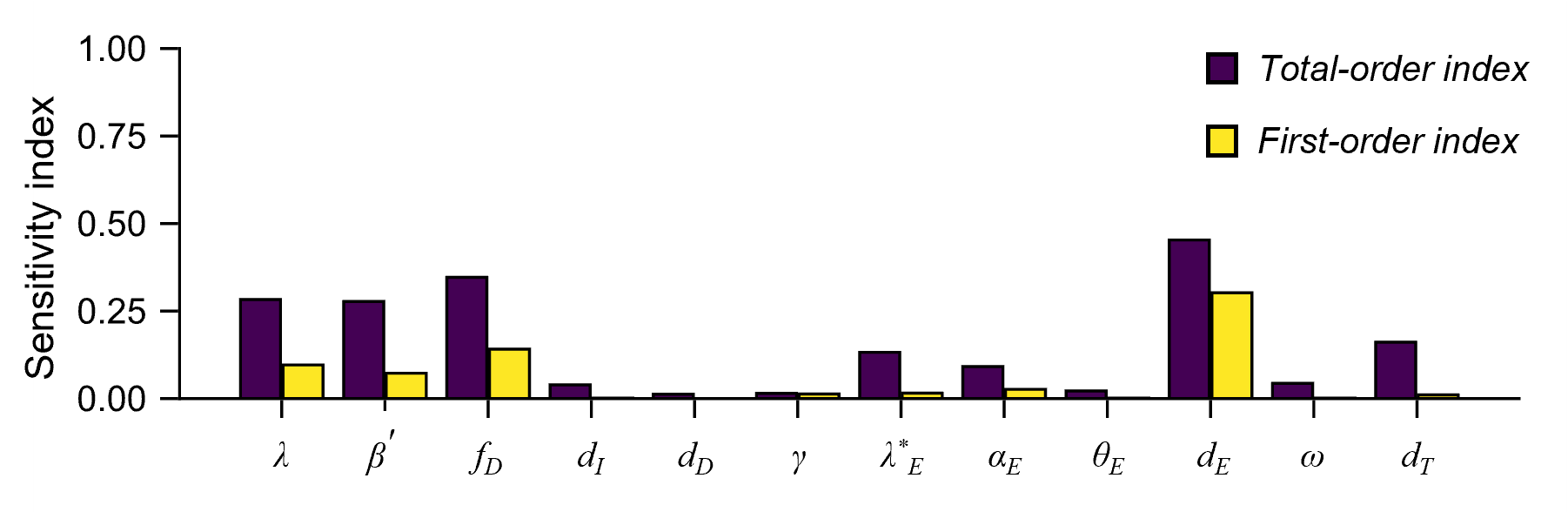

Supplement: S11 Fig — Sensitivity of the set-point viral load predicted by the best-fit model to its parameters estimated using Sobol’s method. (TIF) [file pcbi.1012434.s013.tif]

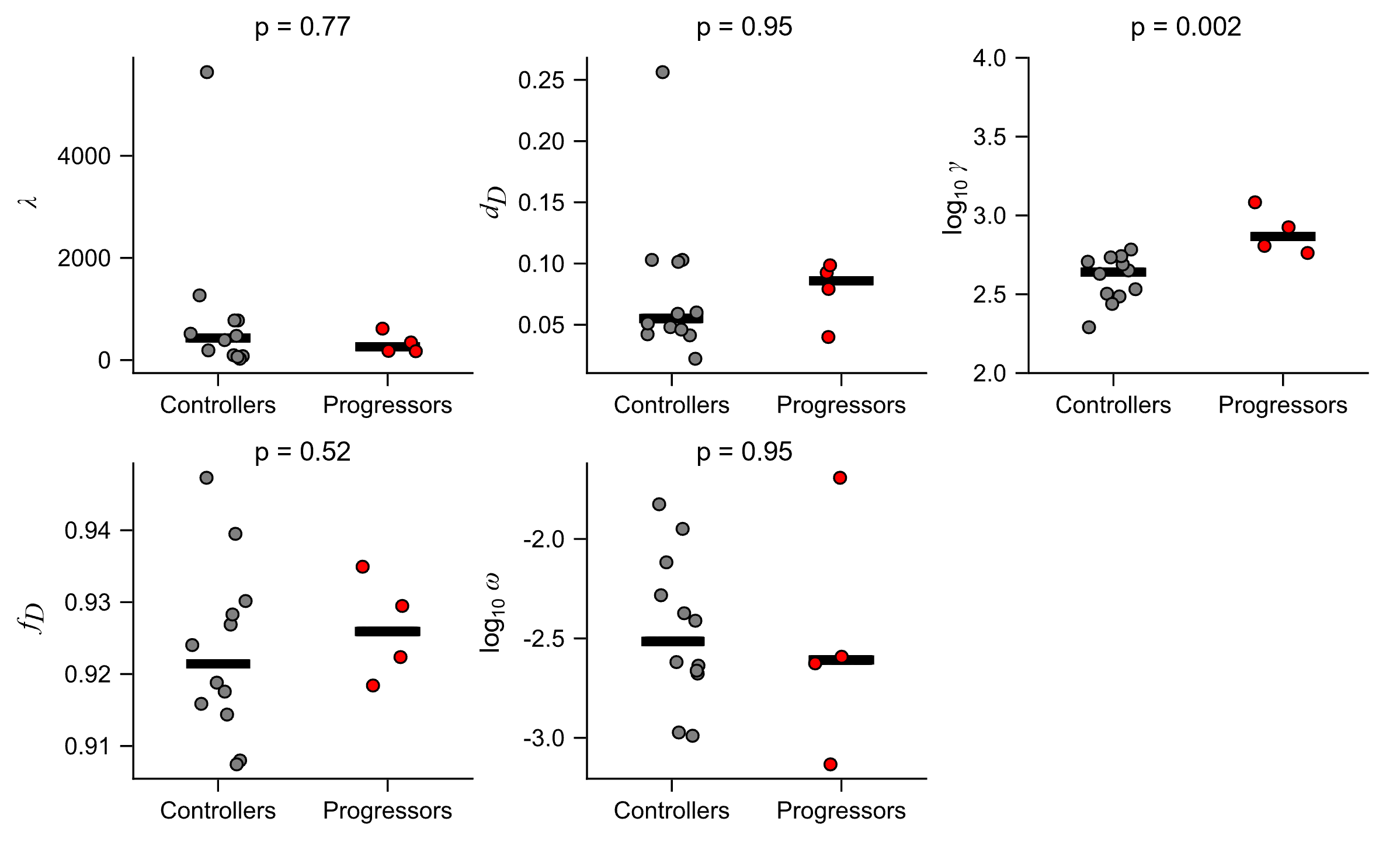

Supplement: S12 Fig — Parameters estimated for all the individuals are grouped based on their control status—controllers vs. progressors—and compared. Presented here are five parameters (λ, dD, αE, fD and log10ω). The others are in Fig 3. Mann-Whitney U test was used to estimate the significance levels. (TIF) [file pcbi.1012434.s014.tif]

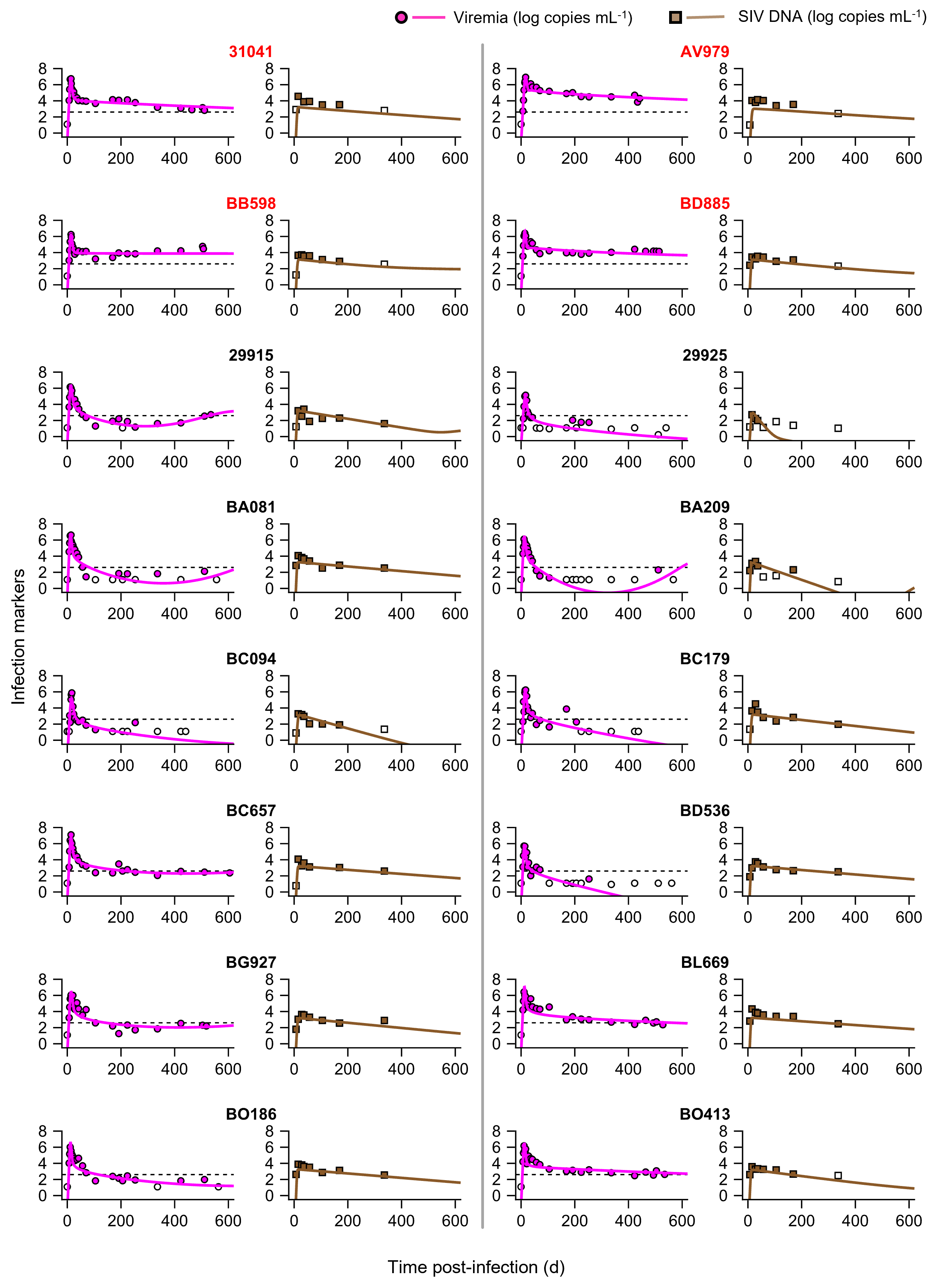

Supplement: S13 Fig — Model predictions (lines) from simultaneous fitting of model #10 (Methods; S1 Table) to all the two virological datasets (symbols), namely, viremia (magenta) and SIV DNA (brown). Macaques highlighted in red are progressors while the rest are controllers. Empty symbols are observations below the limit of detection. The parameter estimates resulting in these fits are in S12 Table. (TIF) [file pcbi.1012434.s015.tif]

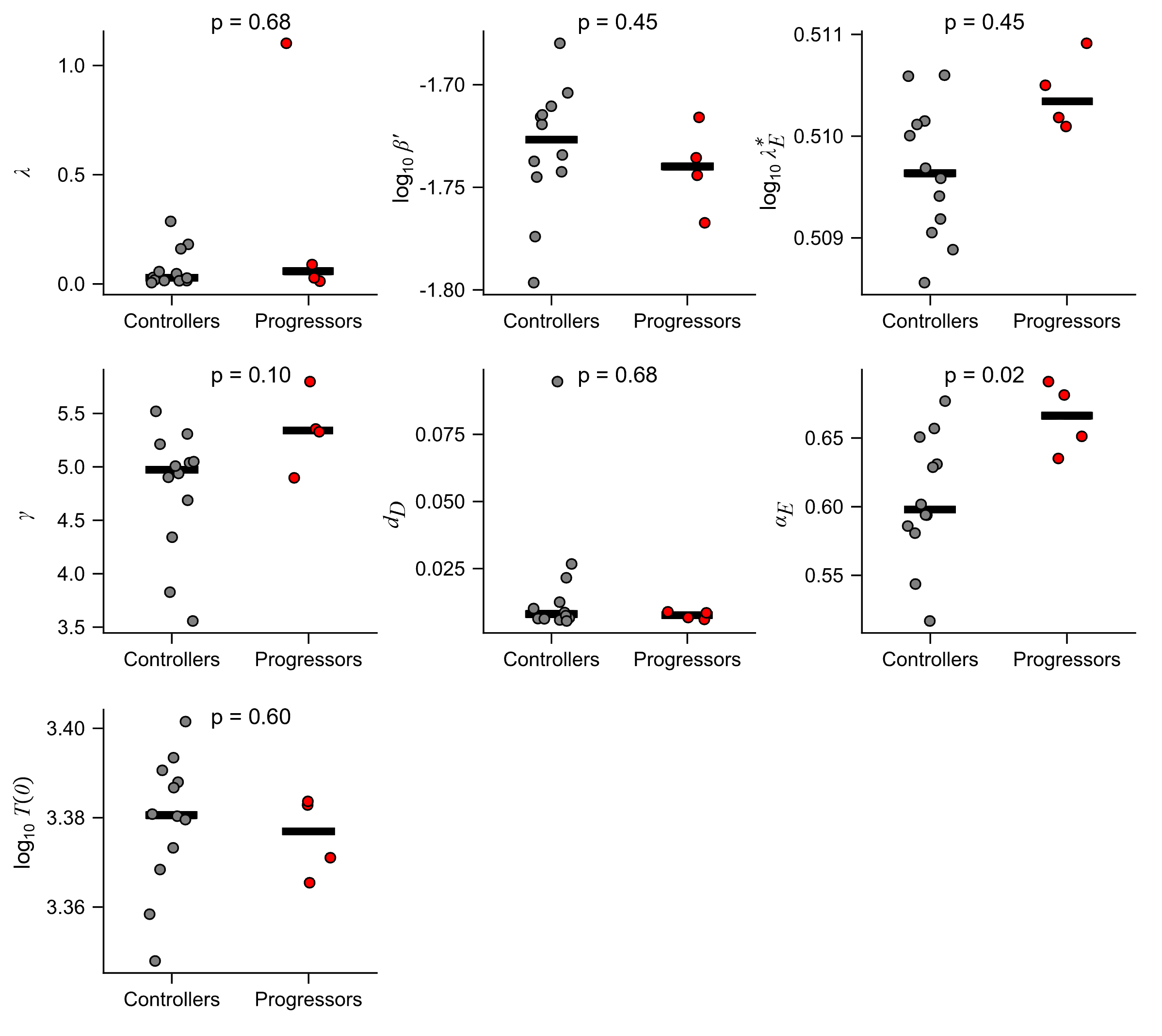

Supplement: S14 Fig — Parameters estimated for all the individuals are grouped based on their control status—controllers vs. progressors—and compared. Mann-Whitney U test was used to estimate the significance levels. (TIF) [file pcbi.1012434.s016.tif]

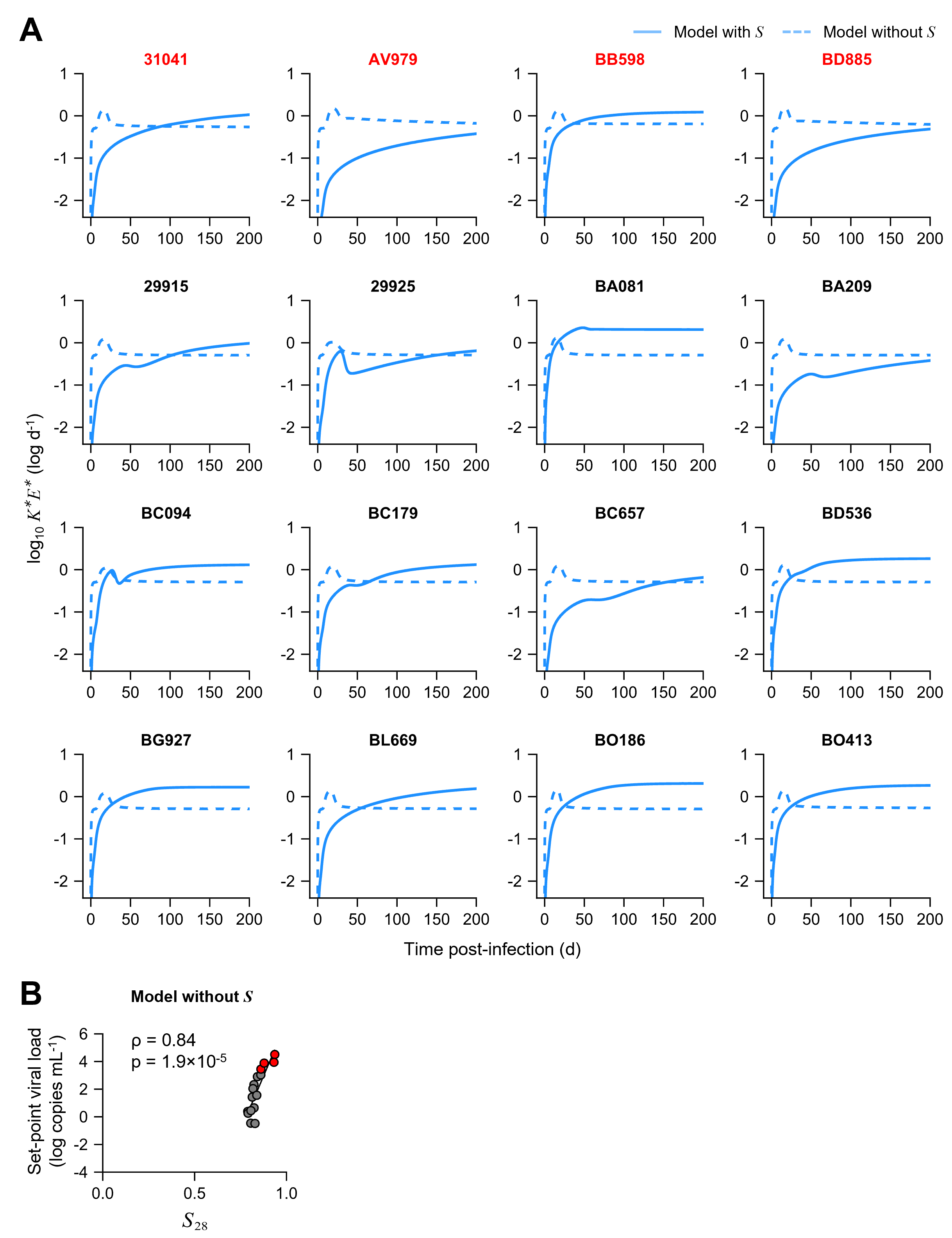

Supplement: S15 Fig — (A) Effector response dynamics of CD8 T-cells, given by K*E*, predicted for the macaques by the best-fit model (solid) and model #10, which does not incorporate suppressive capacity measurements for fitting (dashed). (B) Correlation plot between S28 and set-point viral load as predicted by model #10. Gray symbols are controllers, while red symbols are progressors. Spearman’s ρ was calculated for assessing the correlation. Note that here the set-point viral load increases with S28, which is the opposite of what is expected. (TIF) [file pcbi.1012434.s017.tif]

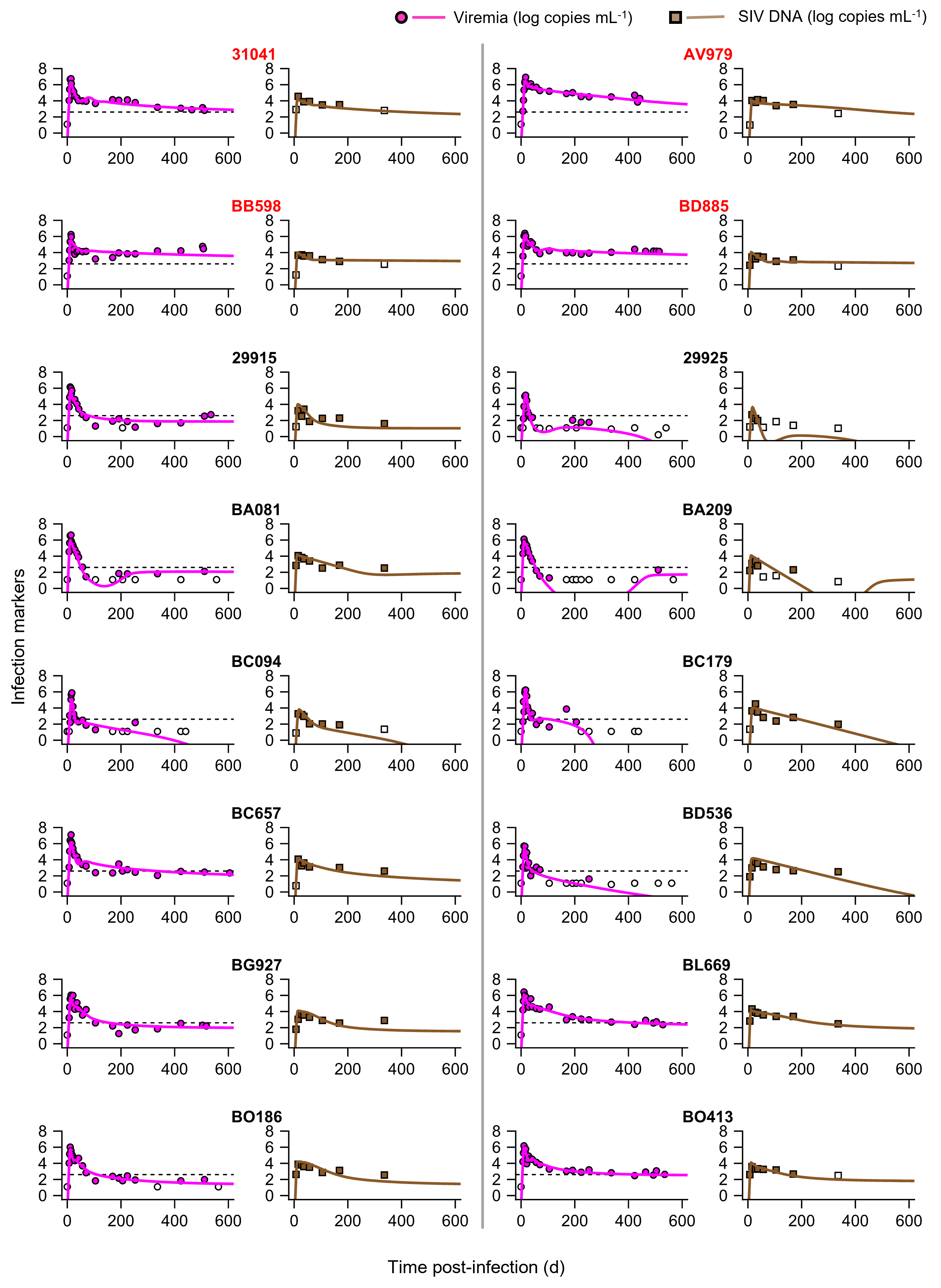

Supplement: S16 Fig — Model predictions (lines) from simultaneous fitting of model #11 (Methods; S1 Table) to all the two virological datasets (symbols), namely, viremia (magenta) and SIV DNA (brown). Macaques highlighted in red are progressors while the rest are controllers. Empty symbols are observations below the limit of detection. The parameter estimates resulting in these fits are in S13 Table. (TIF) [file pcbi.1012434.s018.tif]

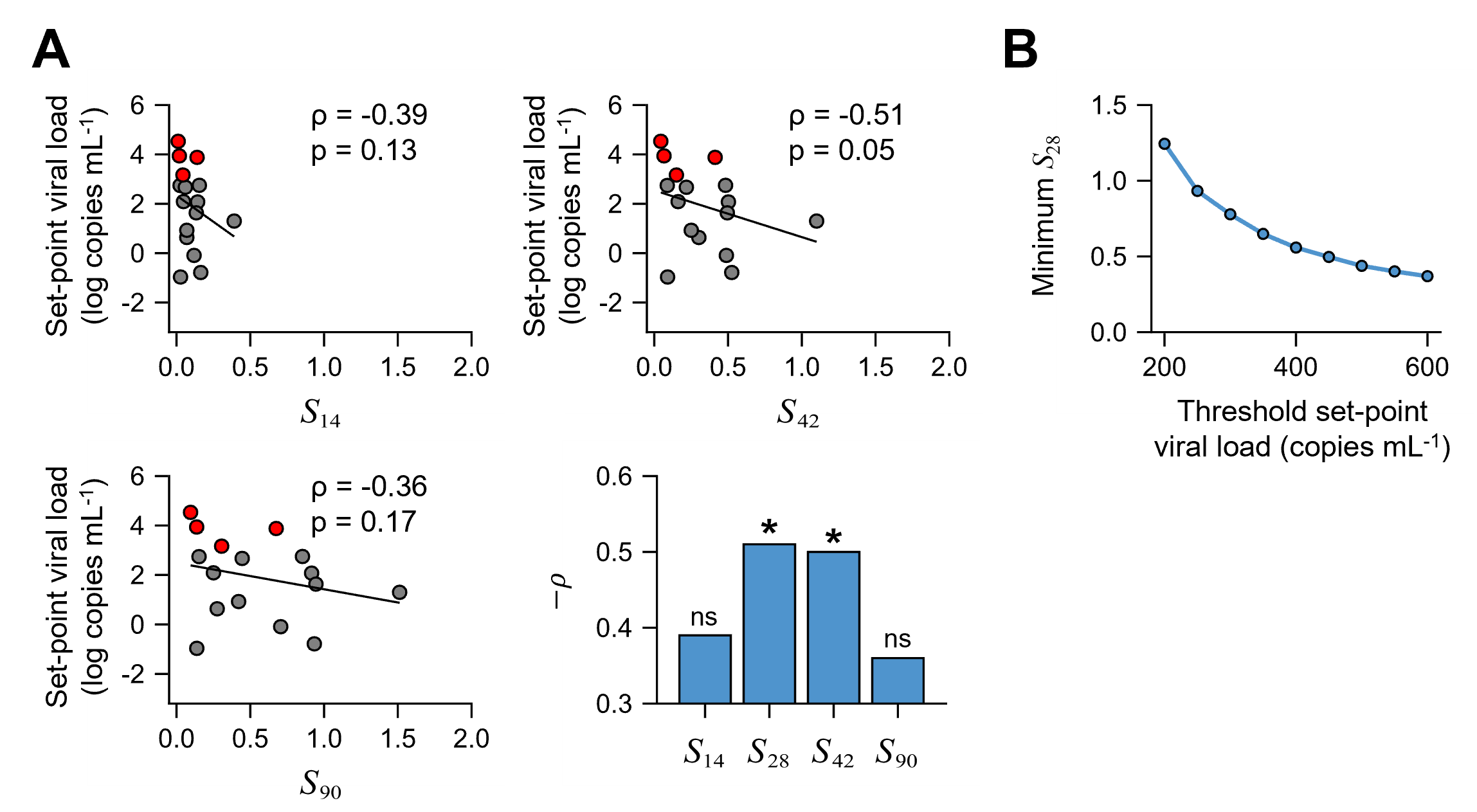

Supplement: S17 Fig — (A) Sensitivity to duration for evaluating the early CD8 T-cell responses. Correlation between set-point viral load and AUC of suppressive capacity averaged over 14, 42 and 90 days post infection, respectively, for the 16 macaques. Gray symbols are controllers, while red symbols are progressors. The bar plot at the bottom right presents the predicted correlation between set-point viral load and the time-averaged area-under-the-curve of S estimated for different durations. Asterisks represent significant correlations with p<0.05; ns: not significant. (B) Minimum S28 required for control increases with a stricter definition of control. The minimum S28 estimated to be required for 95% likelihood of control as a function of the threshold viral load for control. Spearman’s ρ was calculated for assessing the correlations. (TIF) [file pcbi.1012434.s019.tif]

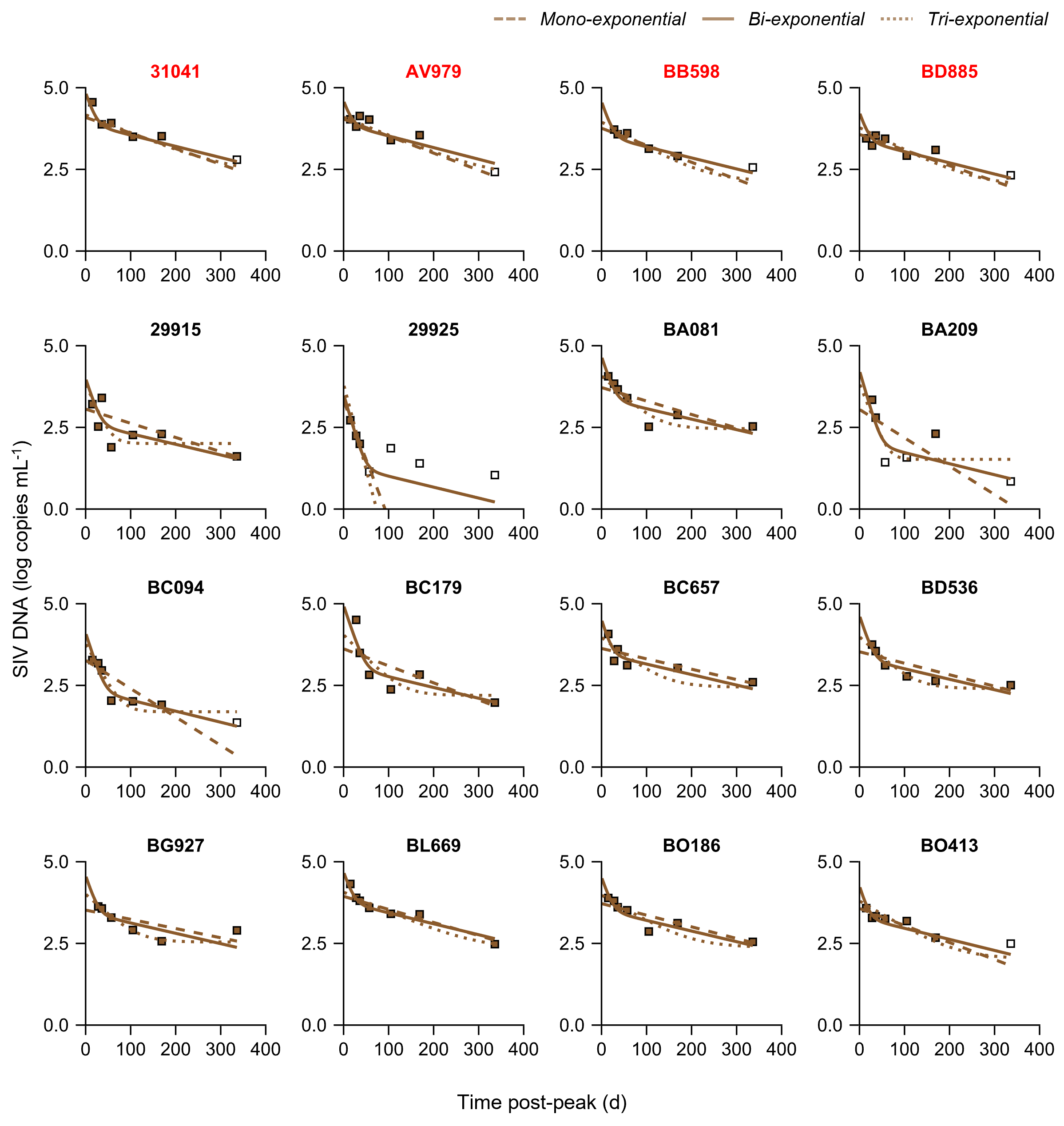

Supplement: S18 Fig — Mono- (dashed), bi- (solid), and tri-exponential (dotted) curves are fitted to longitudinal SIV DNA data post the peak in the measurements. Empty symbols are below detection limit. Data were fit in Monolix (Methods; main text). The bi-exponential curve explained the data best (BICs: 164.48 for the mono-exponential curve; 140.36 for the bi-exponential curve; and 171.13 for the tri-exponential curve). (TIF) [file pcbi.1012434.s020.tif]

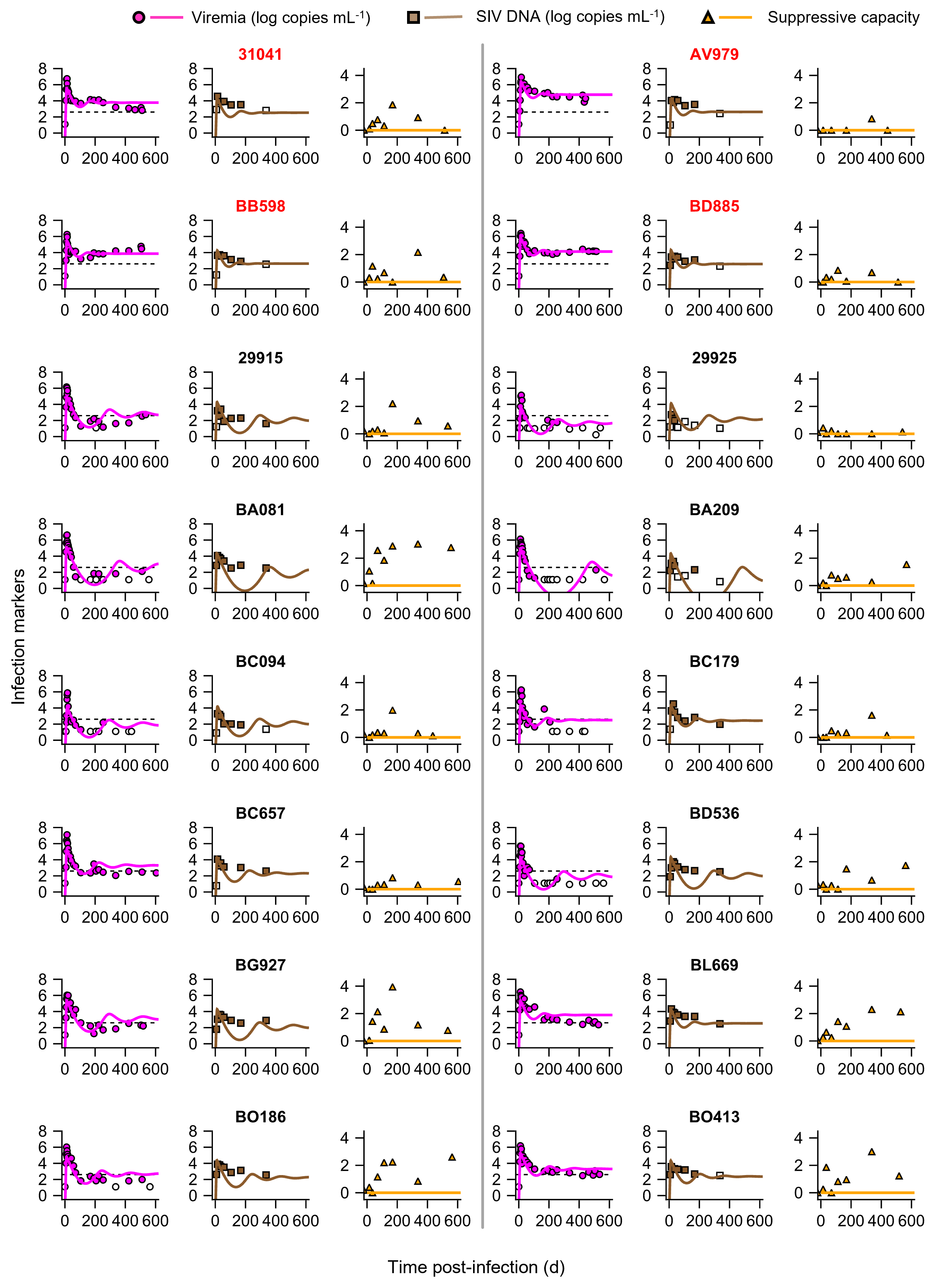

Supplement: S19 Fig — Model predictions (lines) from simultaneous fitting of model #12 (Methods; S1 Table) to all the two virological datasets (symbols), namely, viremia (magenta) and SIV DNA (brown). Macaques highlighted in red are progressors while the rest are controllers. Empty symbols are observations below the limit of detection. The parameter estimates resulting in these fits are in S15 Table. (TIF) [file pcbi.1012434.s021.tif]
