# Supplementary material for: Antiviral capacity of the early CD8 T-cell response is predictive of natural control of SIV infection: Learning in vivo dynamics using ex vivo data
Source: PLoS Comput Biol. 2024 Sep 10;20(9):e1012434. doi: 10.1371/journal.pcbi.1012434 (PMC11414924; doi:10.1371/journal.pcbi.1012434)
Supplement: S1 Table — Every model fit to the data is summarized, comparing the BICs of fits. *These fits do not include suppressive capacity datasets and hence cannot be compared with other models directly. S14 Table presents the comparison of BIC of these models with that of the main model after eliminating the contribution from the suppressive capacity data for the latter. (DOCX) [file pcbi.1012434.s022.docx]

| **Model** | **Description** | **BIC** | **Observation** | **Figure** | **Parameters** |
| --- | --- | --- | --- | --- | --- |
| 1 | Best-fit model (equations (1) – (10) in main text) | 1505 | Explains the data best | Fig. 2; Fig. S10 | Table 1; Table S10 |
| 2 | Model with exhaustion dependent on instantaneous antigen level (Equation (S1)) | 1546 | Model fits do not capture the data any better than the main model | Fig. S2 | Table S2 |
| 3 | Model with exhaustion dependent on cumulative antigen level; Hill coefficient *n* = 1 (Equation (S2)) | 1563 | Model fits do not capture the data any better than the main model | Fig. S3 | Table S3 |
| 4 | Model with exhaustion dependent on cumulative antigen level; Hill coefficient *n* = 4 (Equation (S2)) | 1557 | Model fits do not capture the data any better than the main model | Fig. S4 | Table S4 |
| 5 | Main model with added antigen-dependent effector CD8 T-cell recruitment | 1544 | Model fits do not capture the data any better than the main model | Fig. S5 | Table S5 |
| 6 | Main model fitted with constant  | 1796 | Suppressive capacity measurements are captured worse than the main model | Fig. S6 | Table S6 |
| 7 | Main model fitted with  starting with  and exponentially decreasing with time to  | 1579 | Model fits capture the data worse than the main model | Fig. S7 | Table S7 |
| 8 | Model fitted with $k$ rising to peak before saturating to a lower value with time | 1509 | Model fits do not capture the data any better than the main model | Fig. S8 | Table S8 |
| 9 | Model incorporating non-cytolytic effects of CD8 T-cells | 1521 | Model fits do not capture the data any better than the main model | Fig. S9 | Table S9 |
| 10 | Main model fitted with constant $k$ without the suppressive capacity data | –* | Effector cell dynamics are indistinguishable between controllers and progressors | Fig. S13 | Table S12 |
| 11 | Main model fitted without the suppressive capacity data | –* | Parameter estimates have large confidence intervals | Fig. S16 | Table S13 |
| 12 | Main model fitted with constant $k$ and no $D$ compartment | 1925 | Model fits capture the data worse than the main model | Fig. S19 | Table S15 |

**Table S1: Comparison of different models fitted to the data.** Every model fit to the data is summarized, comparing the BICs of fits. *These fits do not include suppressive capacity datasets and hence cannot be compared with other models directly. Table S14 presents the comparison of BIC of these models with that of the main model after eliminating the contribution from the suppressive capacity data for the latter.
