# Supplementary material for: Antiviral capacity of the early CD8 T-cell response is predictive of natural control of SIV infection: Learning in vivo dynamics using ex vivo data
Source: PLoS Comput Biol. 2024 Sep 10;20(9):e1012434. doi: 10.1371/journal.pcbi.1012434 (PMC11414924; doi:10.1371/journal.pcbi.1012434)
Supplement: S2 Table — The fixed and random effects of each parameter is provided along with respective percent standard errors in parentheses. In addition to the parameters fixed in model #1, fD is fixed to 0.95 and θX is fixed to 5 cells mL-1 [1, 2]. (DOCX) [file pcbi.1012434.s023.docx]

| **Parameter (Units)** | **Fixed effect** | **Random effect** |
| --- | --- | --- |
| $\lambda$ (cells mL^-1^ d^-1^) | 210 (27) | 0.76 (2.89) |
| $\log_{10}\beta'$ (log mL cells^-1^ d^-1^) | -2.39 (2.12) | 0.06 (64) |
| $f_{D}$ | 0.95 | - |
| $\log_{10}\lambda_{E}^{*}$ (log d^-2^) | -0.03 (216) | 0.22 (24.7) |
| $d_{I}$ (d^-1^) | 0.10 | - |
| $d_{D}$ (d^-1^) | 0.06 (27.8) | 0.91 (24.5) |
| $\gamma$ (cells^-1^) | 517 (32.7) | 1.13 (24.1) |
| $\alpha_{E}$ (d^-1^) | 1.00 | - |
| $\theta_{E}$ (cells mL^-1^) | 0.10 | - |
| $\alpha_{R}$ (d^-1^) | 0.02 (217) | 0.94 (302) |
| $\theta_{X}$ (cells mL^-1^) | 5.00 | - |
| $d_{E}$ (d^-1^) | 1.00 | - |
| $\log_{10}\omega$ (log d^-1^) | -2.15 (5.89) | 0.37 (25.5) |
| $\log_{10}T(0)$ (log cells mL^-1^) | 3.94 (1.24) | 0.01 (1.1) |

**Table S2:** **Population parameter estimates for model #2.** The fixed and random effects of each parameter is provided along with respective percent standard errors in parentheses. In addition to the parameters fixed in model #1, $f_{D}$ is fixed to 0.95 and $\theta_{X}$ is fixed to 5 cells mL^-1^ [1, 2].

**References**

1. Conway JM, Perelson AS. Post-treatment control of HIV infection. Proc Natl Acad Sci U S A. 2015;112(17):5467-72. Epub 20150413. doi: 10.1073/pnas.1419162112. PubMed PMID: 25870266; PubMed Central PMCID: PMCPMC4418889.

2. Wang S, Hottz P, Schechter M, Rong L. Modeling the Slow CD4+ T Cell Decline in HIV-Infected Individuals. PLoS Comput Biol. 2015;11(12):e1004665. Epub 20151228. doi: 10.1371/journal.pcbi.1004665. PubMed PMID: 26709961; PubMed Central PMCID: PMCPMC4692447.
