# Supplementary material for: Antiviral capacity of the early CD8 T-cell response is predictive of natural control of SIV infection: Learning in vivo dynamics using ex vivo data
Source: PLoS Comput Biol. 2024 Sep 10;20(9):e1012434. doi: 10.1371/journal.pcbi.1012434 (PMC11414924; doi:10.1371/journal.pcbi.1012434)
Supplement: S3 Table — The Hill coefficient for the exhaustion rate, n = 1. The fixed and random effects of each parameter is provided along with respective percent standard errors in parentheses. In addition to the parameters fixed in model #1, fD is fixed to 0.95, ϕ is fixed to 2 and κ is fixed to 1 d-1 [1–3]. (DOCX) [file pcbi.1012434.s024.docx]

| **Parameter (Units)** | **Fixed effect** | **Random effect** |
| --- | --- | --- |
| $\lambda$ (cells mL^-1^ d^-1^) | 8.91×10^3^ (38.4) | 1.13 (21.1) |
| $\log_{10}\beta'$ (log mL cells^-1^ d^-1^) | -4.10 (3.18) | 0.02 (487) |
| $f_{D}$ | 0.95 | - |
| $\log_{10}\lambda_{E}^{*}$ (log d^-2^) | -0.189 (277) | 0.27 (106) |
| $d_{I}$ (d^-1^) | 0.10 | - |
| $d_{D}$ (d^-1^) | 1.72 (78.5) | 0.75 (77.2) |
| $\gamma$ (cells^-1^) | 21.3 (62) | 0.78 (35.9) |
| $\alpha_{E}$ (d^-1^) | 1.66 (20.8) | 0.02 (725) |
| $\theta_{E}$ (cells mL^-1^) | 0.10 | - |
| $\xi$ (d^-1^) | 1.83 (223) | 0.17 (127) |
|  | 2.00 | - |
| $d_{E}$ (d^-1^) | 1.00 | - |
| $\kappa$ (d^-1^) | 1.00 | - |
| $d_{Q}$ (d^-1^) | 0.52 (278) | 0.19 (425) |
| $\log_{10}\omega$ (log d^-1^) | -2.48 (14.6) | 0.45 (31.3) |
| $\log_{10}T(0)$ (log cells mL^-1^) | 5.64 (2.31) | 0.01 (74.5) |

**Table S3:** **Population parameter estimates for model #3.** The Hill coefficient for the exhaustion rate, n=1. The fixed and random effects of each parameter is provided along with respective percent standard errors in parentheses. In addition to the parameters fixed in model #1, $f_{D}$ is fixed to 0.95, is fixed to 2 and $\kappa$ is fixed to 1 d^-1^ [1-3].

**References**

1. Conway JM, Perelson AS. Post-treatment control of HIV infection. Proc Natl Acad Sci U S A. 2015;112(17):5467-72. Epub 20150413. doi: 10.1073/pnas.1419162112. PubMed PMID: 25870266; PubMed Central PMCID: PMCPMC4418889.

2. Johnson PL, Kochin BF, McAfee MS, Stromnes IM, Regoes RR, Ahmed R, et al. Vaccination alters the balance between protective immunity, exhaustion, escape, and death in chronic infections. J Virol. 2011;85(11):5565-70. Epub 20110316. doi: 10.1128/JVI.00166-11. PubMed PMID: 21411537; PubMed Central PMCID: PMCPMC3094965.

3. Wang S, Hottz P, Schechter M, Rong L. Modeling the Slow CD4+ T Cell Decline in HIV-Infected Individuals. PLoS Comput Biol. 2015;11(12):e1004665. Epub 20151228. doi: 10.1371/journal.pcbi.1004665. PubMed PMID: 26709961; PubMed Central PMCID: PMCPMC4692447.
