# Supplementary material for: Antiviral capacity of the early CD8 T-cell response is predictive of natural control of SIV infection: Learning in vivo dynamics using ex vivo data
Source: PLoS Comput Biol. 2024 Sep 10;20(9):e1012434. doi: 10.1371/journal.pcbi.1012434 (PMC11414924; doi:10.1371/journal.pcbi.1012434)
Supplement: S4 Table — The Hill coefficient for the exhaustion rate, n = 4. The fixed and random effects of each parameter is provided along with respective percent standard errors in parentheses. In addition to the parameters fixed in model #1, fD is fixed to 0.95, ϕ is fixed to 2 and κ is fixed to 1 d-1 [1–3]. (DOCX) [file pcbi.1012434.s025.docx]

| **Parameter (Units)** | **Fixed effect** | **Random effect** |
| --- | --- | --- |
| $\lambda$ (cells mL^-1^ d^-1^) | 3.41×10^3^ (135) | 1.17 (25.5) |
| $\log_{10}\beta'$ (log mL cells^-1^ d^-1^) | -3.66 (14.2) | 0.03 (333) |
| $f_{D}$ | 0.95 | - |
| $\log_{10}\lambda_{E}^{*}$ (log d^-2^) | 0.29 (102) | 0.16 (106) |
| $d_{I}$ (d^-1^) | 0.10 | - |
| $d_{D}$ (d^-1^) | 0.52 (176) | 0.76 (114) |
| $\gamma$ (cells^-1^) | 63.6 (129) | 0.74 (48.4) |
| $\alpha_{E}$ (d^-1^) | 1.23 (88.8) | 0.19 (212) |
| $\theta_{E}$ (cells mL^-1^) | 0.10 | - |
| $\xi$ (d^-1^) | 0.99 (174) | 0.28 (205) |
|  | 2.00 | - |
| $d_{E}$ (d^-1^) | 1.00 | - |
| $\kappa$ (d^-1^) | 1.00 | - |
| $d_{Q}$ (d^-1^) | 0.01 (722) | 1.08 (536) |
| $\log_{10}\omega$ (log d^-1^) | -2.37 (12) | 0.40 (28.3) |
| $\log_{10}T(0)$ (log cells mL^-1^) | 5.22 (10.5) | 0.01 (46.3) |

**Table S4:** **Population parameter estimates for model #4.** The Hill coefficient for the exhaustion rate, n=4. The fixed and random effects of each parameter is provided along with respective percent standard errors in parentheses. In addition to the parameters fixed in model #1, $f_{D}$ is fixed to 0.95, is fixed to 2 and $\kappa$ is fixed to 1 d^-1^ [1-3].

**References**

1. Conway JM, Perelson AS. Post-treatment control of HIV infection. Proc Natl Acad Sci U S A. 2015;112(17):5467-72. Epub 20150413. doi: 10.1073/pnas.1419162112. PubMed PMID: 25870266; PubMed Central PMCID: PMCPMC4418889.

2. Johnson PL, Kochin BF, McAfee MS, Stromnes IM, Regoes RR, Ahmed R, et al. Vaccination alters the balance between protective immunity, exhaustion, escape, and death in chronic infections. J Virol. 2011;85(11):5565-70. Epub 20110316. doi: 10.1128/JVI.00166-11. PubMed PMID: 21411537; PubMed Central PMCID: PMCPMC3094965.

3. Wang S, Hottz P, Schechter M, Rong L. Modeling the Slow CD4+ T Cell Decline in HIV-Infected Individuals. PLoS Comput Biol. 2015;11(12):e1004665. Epub 20151228. doi: 10.1371/journal.pcbi.1004665. PubMed PMID: 26709961; PubMed Central PMCID: PMCPMC4692447.
