# Supplementary material for: Antiviral capacity of the early CD8 T-cell response is predictive of natural control of SIV infection: Learning in vivo dynamics using ex vivo data
Source: PLoS Comput Biol. 2024 Sep 10;20(9):e1012434. doi: 10.1371/journal.pcbi.1012434 (PMC11414924; doi:10.1371/journal.pcbi.1012434)
Supplement: S5 Table — The fixed and random effects of each parameter is provided along with respective percent standard errors in parentheses. In addition to the parameters fixed in model #1, fD is fixed to 0.95 [1]. (DOCX) [file pcbi.1012434.s026.docx]

| **Parameter (Units)** | **Fixed effect** | **Random effect** |
| --- | --- | --- |
| $\lambda$ (cells mL^-1^ d^-1^) | 3.13×10^3^ (72.6) | 1.20 (22.8) |
| $\log_{10}\beta'$ (log mL cells^-1^ d^-1^) | -3.65 (3.14) | 0.02 (248) |
| $f_{D}$ | 0.95 | - |
| $\log_{10}\lambda_{E}^{*}$ (log d^-2^) | -0.23 (727) | 0.29 (348) |
| $d_{I}$ (d^-1^) | 0.10 | - |
| $d_{D}$ (d^-1^) | 0.45 (51.8) | 0.62 (86) |
| $\gamma$ (cells^-1^) | 65.90 (46.5) | 0.77 (38.4) |
| $\rho_{E}$ (cells mL^-1^ d^-2^) | 0.25 (1.67×10^3^) | 1.07 (632) |
| $\alpha_{E}$ (d^-1^) | 0.66 (124) | 0.21 (256) |
| $\theta_{E}$ (cells mL^-1^) | 0.10 | - |
| $d_{E}$ (d^-1^) | 1.00 | - |
| $\log_{10}\omega$ (log d^-1^) | -2.44 (8.22) | 0.42 (31.1) |
| $\log_{10}T(0)$ (log cells mL^-1^) | 5.19 (2.17) | 0.01 (44) |

**Table S5:** **Population parameter estimates for model #5.** The fixed and random effects of each parameter is provided along with respective percent standard errors in parentheses. In addition to the parameters fixed in model #1, $f_{D}$ is fixed to 0.95 [1].

**References**

1. Wang S, Hottz P, Schechter M, Rong L. Modeling the Slow CD4+ T Cell Decline in HIV-Infected Individuals. PLoS Comput Biol. 2015;11(12):e1004665. Epub 20151228. doi: 10.1371/journal.pcbi.1004665. PubMed PMID: 26709961; PubMed Central PMCID: PMCPMC4692447.
