# Supplementary material for: Antiviral capacity of the early CD8 T-cell response is predictive of natural control of SIV infection: Learning in vivo dynamics using ex vivo data
Source: PLoS Comput Biol. 2024 Sep 10;20(9):e1012434. doi: 10.1371/journal.pcbi.1012434 (PMC11414924; doi:10.1371/journal.pcbi.1012434)
Supplement: S6 Table — The fixed and random effects of each parameter is provided along with respective percent standard errors in parentheses. Similar to the best-fit model (Table 1), parameters dI, θE and dE were fixed. (DOCX) [file pcbi.1012434.s027.docx]

| **Parameter (Units)** | **Fixed effect** | **Random effect** |
| --- | --- | --- |
| $\lambda$ (cells mL^-1^ d^-1^) | 22×10^-3^ (115) | 2.02 (50.6) |
| $\log_{10}\beta'$ (log mL cells^-1^ d^-1^) | -2.03 (0.12) | - |
| $f_{D}$ | 0.9 (0.67) | 0.2 (29.3) |
| $\log_{10}\lambda_{E}^{*}$ (log d^-2^) | -0.22 (35.3) | 0.19 (45.4) |
| $d_{I}$ (d^-1^) | 0.10 | - |
| $d_{D}$ (d^-1^) | 5.7×10^-3^ (55.2) | 1.35 (36.1) |
| $\gamma$ (cells^-1^) | 44.62×10^3^ (37.4) | 1.36 (20.3) |
| $\alpha_{E}$ (d^-1^) | 0.57 (8.01) | 0.2 (44.3) |
| $\theta_{E}$ (cells mL^-1^) | 0.10 | - |
| $d_{E}$ (d^-1^) | 1.00 | - |
| $\log_{10}T(0)$ (log cells mL^-1^) | 3.4 (0.06) | - |

**Table S6 Population parameter estimates for model #6.** The fixed and random effects of each parameter is provided along with respective percent standard errors in parentheses. Similar to the best-fit model (Table 1), parameters $d_{I}$, $\theta_{E}$ and $d_{E}$ were fixed.
