# Supplementary material for: Antiviral capacity of the early CD8 T-cell response is predictive of natural control of SIV infection: Learning in vivo dynamics using ex vivo data
Source: PLoS Comput Biol. 2024 Sep 10;20(9):e1012434. doi: 10.1371/journal.pcbi.1012434 (PMC11414924; doi:10.1371/journal.pcbi.1012434)
Supplement: S7 Table — The fixed and random effects of each parameter is provided along with respective percent standard errors in parentheses. Similar to the best-fit model (Table 1), parameters dI, θE and dE were fixed. In addition, log10 ω was fixed to -2.50 from Table 1. (DOCX) [file pcbi.1012434.s028.docx]

| **Parameter (Units)** | **Fixed effect** | **Random effect** |
| --- | --- | --- |
| $\lambda$ (cells mL^-1^ d^-1^) | 353.82 (43.7) | 1.3 (22.1) |
| $\log_{10}\beta'$ (log mL cells^-1^ d^-1^) | -3.08 (5.11) | 0.05 (72.8) |
| $f_{D}$ | 0.7 (7.2) | 0.12 (86) |
| $\log_{10}\lambda_{E}^{*}$ (log d^-2^) | -2.42 (6.97) | 0.38 (27.4) |
| $d_{I}$ (d^-1^) | 0.10 | - |
| $d_{D}$ (d^-1^) | 0.08 (31.2) | 0.92 (27.7) |
| $\gamma$ (cells^-1^) | 65.76 (36.1) | 1.22 (23.8) |
| $\alpha_{E}$ (d^-1^) | 1.06 (1.64) | 0.06 (24.3) |
| $\theta_{E}$ (cells mL^-1^) | 0.10 | - |
| $d_{E}$ (d^-1^) | 1.00 | - |
| $\frac{k_{i}}{k_{f}}-1$ | 7.4×10^-3^ (47.2) | 2.06 (105) |
| $\log_{10}\omega$ (log d^-1^) | -2.50 | - |
| $\log_{10}T(0)$ (log cells mL^-1^) | 3.85 (2.65) | 0.01 (120) |

**Table S7 Population parameter estimates for model #7.** The fixed and random effects of each parameter is provided along with respective percent standard errors in parentheses. Similar to the best-fit model (Table 1), parameters $d_{I}$, $\theta_{E}$ and $d_{E}$ were fixed. In addition, $\log_{10}\omega$ was fixed to -2.50 from Table 1.
