# Supplementary material for: Antiviral capacity of the early CD8 T-cell response is predictive of natural control of SIV infection: Learning in vivo dynamics using ex vivo data
Source: PLoS Comput Biol. 2024 Sep 10;20(9):e1012434. doi: 10.1371/journal.pcbi.1012434 (PMC11414924; doi:10.1371/journal.pcbi.1012434)
Supplement: S8 Table — The fixed and random effects of each parameter is provided along with respective percent standard errors in parentheses. Similar to the best-fit model (Table 1), parameters dI, θE and dE were fixed. In addition, log10 β’ and log10 T(0) were fixed using values from Table 1. (DOCX) [file pcbi.1012434.s029.docx]

| **Parameter (Units)** | **Fixed effect** | **Random effect** |
| --- | --- | --- |
| $\lambda$ (cells mL^-1^ d^-1^) | 361 (39.6) | 1.39 (21.6) |
| $\log_{10}\beta'$ (log mL cells^-1^ d^-1^) | -2.84 | - |
| $f_{D}$ | 0.92 (0.37) | 0.17 (20) |
| $\log_{10}\lambda_{E}^{*}$ (log d^-2^) | 0.15 (102) | 0.14 (144) |
| $d_{I}$ (d^-1^) | 0.1 | - |
| $d_{D}$ (d^-1^) | 0.07 (26.4) | 0.69 (29.6) |
| $\gamma$ (cells^-1^) | 436 (38.7) | 0.67 (73.7) |
| $\alpha_{E}$ (d^-1^) | 0.53 (42.8) | 0.29 (85.3) |
| $\theta_{E}$ (cells mL^-1^) | 0.1 | - |
| $d_{E}$ (d^-1^) | 1.0 | - |
| $\mu$ | 0 (0) | 197 (189) |
| $\log_{10}\omega_{1}$ (log d^-1^) | -2.4 (6.42) | 0.45 (30.9) |
| $\log_{10}\omega_{2}$ (log d^-1^) | -3.23 (391) | 0.87 (1550) |
| $\log_{10}T(0)$ (log cells mL^-1^) | 4.21 | - |

**Table S8 Population parameter estimates for model #8.** The fixed and random effects of each parameter is provided along with respective percent standard errors in parentheses. Similar to the best-fit model (Table 1), parameters $d_{I}$, $\theta_{E}$ and $d_{E}$ were fixed. In addition, $\log_{10}\beta'$ and $\log_{10}T(0)$ were fixed using values from Table 1.
