# Supplementary material for: Antiviral capacity of the early CD8 T-cell response is predictive of natural control of SIV infection: Learning in vivo dynamics using ex vivo data
Source: PLoS Comput Biol. 2024 Sep 10;20(9):e1012434. doi: 10.1371/journal.pcbi.1012434 (PMC11414924; doi:10.1371/journal.pcbi.1012434)
Supplement: S9 Table — The fixed and random effects of each parameter is provided along with respective percent standard errors in parentheses. In addition to the parameters fixed in model #1, fD is fixed to 0.95 [1]. (DOCX) [file pcbi.1012434.s030.docx]

| **Parameter (Units)** | **Fixed effect** | **Random effect** |
| --- | --- | --- |
| $\lambda$ (cells mL^-1^ d^-1^) | 440 (51.1) | 1.41 (20.7) |
| $\log_{10}\beta'$ (log mL cells^-1^ d^-1^) | -2.77 (5.15) | 0.05 (190) |
| $f_{D}$ | 0.95 | - |
| $\log_{10}\lambda_{E}^{*}$ (log d^-2^) | 0.16 (51.4) | 0.17 (53.4) |
| $d_{I}$ (d^-1^) | 0.1 | - |
| $d_{D}$ (d^-1^) | 0.09 (43.4) | 0.58 (34.1) |
| $\gamma$ (cells^-1^) | 480 (50) | 0.74 (37.1) |
| $\eta^{*}$ (mL d^2^ cells^-1^) | 2.83×10^-5^ (500) | 1.19 (176) |
| $\alpha_{E}$ (d^-1^) | 0.55 (29.7) | 0.26 (88.8) |
| $\theta_{E}$ (cells mL^-1^) | 0.1 | - |
| $d_{E}$ (d^-1^) | 1.0 | - |
| $\log_{10}\omega$ (log d^-1^) | -2.44 (6.51) | 0.45 (25.3) |
| $\log_{10}T(0)$ (log cells mL^-1^) | 4.31 (3.36) | 0.01 (136) |

**Table S9:** **Population parameter estimates for model #9.** The fixed and random effects of each parameter is provided along with respective percent standard errors in parentheses. In addition to the parameters fixed in model #1, $f_{D}$ is fixed to 0.95 [1].

**References**

1. Wang S, Hottz P, Schechter M, Rong L. Modeling the Slow CD4+ T Cell Decline in HIV-Infected Individuals. PLoS Comput Biol. 2015;11(12):e1004665. Epub 20151228. doi: 10.1371/journal.pcbi.1004665. PubMed PMID: 26709961; PubMed Central PMCID: PMCPMC4692447.
