# Supplementary material for: Antiviral capacity of the early CD8 T-cell response is predictive of natural control of SIV infection: Learning in vivo dynamics using ex vivo data
Source: PLoS Comput Biol. 2024 Sep 10;20(9):e1012434. doi: 10.1371/journal.pcbi.1012434 (PMC11414924; doi:10.1371/journal.pcbi.1012434)
Supplement: S10 Table — Fixed parameters are dI, θE and dE respectively [1,2], as detailed in Methods of main text. Random effects for log10 β’ and log10T(0) were less than 0.1, and were thus removed, rendering them to be same across macaques. (DOCX) [file pcbi.1012434.s031.docx]

| **Macaque ID** | $\lambda$ | $f_{D}$ | $\log_{10}\lambda_{E}^{*}$ | $d_{D}$ | $\gamma$ | $\alpha_{E}$ | $\log_{10}\omega$ |
| --- | --- | --- | --- | --- | --- | --- | --- |
| 29915 | 95.33 | 0.93 | 0.19 | 0.10 | 340.52 | 0.58 | -2.68 |
| 29925 | 77.18 | 0.95 | 0.18 | 0.26 | 318.82 | 0.86 | -2.64 |
| 31041 | 179.47 | 0.92 | 0.07 | 0.04 | 841.63 | 0.58 | -2.59 |
| AV979 | 344.91 | 0.93 | 0.06 | 0.08 | 1211.71 | 0.59 | -3.13 |
| BA081 | 5638.31 | 0.91 | 0.26 | 0.04 | 447.82 | 0.61 | -1.83 |
| BA209 | 25.91 | 0.92 | 0.31 | 0.05 | 508.71 | 0.50 | -2.99 |
| BB598 | 615.66 | 0.93 | -0.25 | 0.09 | 640.96 | 0.56 | -1.69 |
| BC094 | 391.44 | 0.94 | 0.23 | 0.10 | 306.32 | 0.83 | -2.37 |
| BC179 | 190.44 | 0.93 | 0.42 | 0.04 | 426.20 | 0.54 | -2.66 |
| BC657 | 62.97 | 0.93 | 0.20 | 0.05 | 607.85 | 0.59 | -2.97 |
| BD536 | 515.97 | 0.92 | 0.35 | 0.02 | 195.26 | 0.60 | -2.28 |
| BD885 | 172.81 | 0.92 | -0.23 | 0.10 | 577.93 | 0.55 | -2.63 |
| BG927 | 1266.57 | 0.92 | 0.04 | 0.06 | 488.93 | 0.69 | -2.12 |
| BL669 | 474.54 | 0.92 | 0.14 | 0.06 | 552.07 | 0.68 | -2.62 |
| BO186 | 775.64 | 0.91 | 0.27 | 0.05 | 274.69 | 0.72 | -2.41 |
| BO413 | 776.12 | 0.91 | -0.04 | 0.10 | 542.03 | 0.62 | -1.95 |

**Table S10:** **Individual parameter estimates for the best-fit model.** Fixed parameters are $d_{I}$, $\theta_{E}$ and $d_{E}$ respectively [1, 2], as detailed in Methods of main text. Random effects for $\log_{10}\beta'$ and $\log_{10}T(0)$ were less than 0.1, and were thus removed, rendering them to be same across macaques.

**References**

1. Conway JM, Perelson AS. Post-treatment control of HIV infection. Proc Natl Acad Sci U S A. 2015;112(17):5467-72. Epub 20150413. doi: 10.1073/pnas.1419162112. PubMed PMID: 25870266; PubMed Central PMCID: PMCPMC4418889.

2. De Boer RJ, Perelson AS. Quantifying T lymphocyte turnover. J Theor Biol. 2013;327:45-87. Epub 20130109. doi: 10.1016/j.jtbi.2012.12.025. PubMed PMID: 23313150; PubMed Central PMCID: PMCPMC3640348.
