# Supplementary material for: Antiviral capacity of the early CD8 T-cell response is predictive of natural control of SIV infection: Learning in vivo dynamics using ex vivo data
Source: PLoS Comput Biol. 2024 Sep 10;20(9):e1012434. doi: 10.1371/journal.pcbi.1012434 (PMC11414924; doi:10.1371/journal.pcbi.1012434)
Supplement: S11 Table — The table lists the values used, and the references thereof. CD8 T-cell count in untreated SIV-infected cynomolgus macaques was close to 106 cells mL-1 [5], similar to the levels in HIV-infected humans [6,7]. So, we fixed C0 to 106. (DOCX) [file pcbi.1012434.s032.docx]

| **Parameter** | **Description** | **Value** | **Reference** |
| --- | --- | --- | --- |
| $\hat{\beta}$ | Infection of target cells by virions | 10^-8^ mL cells^-1^ d^-1^ | [1] |
| $\delta$ | Death of virus-producing cells | 1.44 d^-1^ | [2] |
| $\rho$ | Transition from eclipse phase to actively producing virions | 0.36 d^-1^ | Estimated; Fig. S1A |
| $\hat{p}$ | Virus production | 1440 d^-1^ | [2] |
| $c$ | Free virion clearance | 0.35 d^-1^ | [2] |
| $f$ | Fraction of infections resulting in non-productive infections | 0.5 | [3] |
| $\hat{T}_{0}$ | Initial number of CD4 T-cells | 10^6^ cells mL^-1^ | [4] |
| $\hat{V}_{0}$ | Viral inoculum size | 10^2.86^ mL^-1^ | [4] |
| $\hat{C}_{0}$ | Concentration of CD8 T-cells extracted for the *ex vivo* assay | 10^6^ cells mL^-1^ | [4] |
| $C_{0}$ | Concentration of total CD8 T-cells in infected hosts | 10^6^ cells mL^-1^ | [5] |
|  | Basic reproductive ratio of virus in cultures | 14.29 | Estimated |
|  | Fraction of target cells infected by peak viral load in CD4 T-cell culture | 0.93 | Estimated |
| $\tau_{\text{max}}$ | Time point in the CD4 T-cell culture when the viral load peaks | 5.64 d | Estimated |

**Table S11:** **Parameters of the ex vivo model.** The table lists the values used, and the references thereof. CD8 T-cell count in untreated SIV-infected cynomolgus macaques was close to 10^6^ cells mL^-1^ [5], similar to the levels in HIV-infected humans [6, 7]. So, we fixed $C_{0}$ to 10^6^.

**References**

1. Arora P, Dixit NM. Timing the emergence of resistance to anti-HIV drugs with large genetic barriers. PLoS Comput Biol. 2009;5(3):e1000305. Epub 20090313. doi: 10.1371/journal.pcbi.1000305. PubMed PMID: 19282958; PubMed Central PMCID: PMCPMC2643484.

2. Dixit NM, Perelson AS. HIV dynamics with multiple infections of target cells. Proc Natl Acad Sci U S A. 2005;102(23):8198-203. Epub 20050531. doi: 10.1073/pnas.0407498102. PubMed PMID: 15928092; PubMed Central PMCID: PMCPMC1149399.

3. Soto PC, Terry VH, Lewinski MK, Deshmukh S, Beliakova-Bethell N, Spina CA. HIV-1 latency is established preferentially in minimally activated and non-dividing cells during productive infection of primary CD4 T cells. PLoS One. 2022;17(7):e0271674. Epub 20220727. doi: 10.1371/journal.pone.0271674. PubMed PMID: 35895672; PubMed Central PMCID: PMCPMC9328514.

4. Saez-Cirion A, Shin SY, Versmisse P, Barre-Sinoussi F, Pancino G. Ex vivo T cell-based HIV suppression assay to evaluate HIV-specific CD8+ T-cell responses. Nat Protoc. 2010;5(6):1033-41. Epub 20100513. doi: 10.1038/nprot.2010.73. PubMed PMID: 20539279.

5. Benlhassan-Chahour K, Penit C, Dioszeghy V, Vasseur F, Janvier G, Riviere Y, et al. Kinetics of lymphocyte proliferation during primary immune response in macaques infected with pathogenic simian immunodeficiency virus SIVmac251: preliminary report of the effect of early antiviral therapy. J Virol. 2003;77(23):12479-93. doi: 10.1128/jvi.77.23.12479-12493.2003. PubMed PMID: 14610172; PubMed Central PMCID: PMCPMC262554.

6. Cao W, Mehraj V, Kaufmann DE, Li T, Routy JP. Elevation and persistence of CD8 T-cells in HIV infection: the Achilles heel in the ART era. J Int AIDS Soc. 2016;19(1):20697. Epub 20160303. doi: 10.7448/IAS.19.1.20697. PubMed PMID: 26945343; PubMed Central PMCID: PMCPMC4779330.

7. Helleberg M, Kronborg G, Ullum H, Ryder LP, Obel N, Gerstoft J. Course and Clinical Significance of CD8+ T-Cell Counts in a Large Cohort of HIV-Infected Individuals. J Infect Dis. 2015;211(11):1726-34. Epub 20141208. doi: 10.1093/infdis/jiu669. PubMed PMID: 25489001.
