# Supplementary material for: Antiviral capacity of the early CD8 T-cell response is predictive of natural control of SIV infection: Learning in vivo dynamics using ex vivo data
Source: PLoS Comput Biol. 2024 Sep 10;20(9):e1012434. doi: 10.1371/journal.pcbi.1012434 (PMC11414924; doi:10.1371/journal.pcbi.1012434)
Supplement: S12 Table — The fixed and random effects of each parameter are provided along with respective percent standard errors in parentheses. In addition to the parameters fixed in the best-fit model, fD is fixed to 0.95 [1]. (DOCX) [file pcbi.1012434.s033.docx]

| **Parameter (Units)** | **Fixed effect** | **Random effect** |
| --- | --- | --- |
| $\lambda$ (cells mL^-1^ d^-1^) | 0.03 (194) | 2.00 (93.3) |
| $\log_{10}\beta'$ (log mL cells^-1^ d^-1^) | -1.73 (3.46) | 0.04 (84.2) |
| $f_{D}$ | 0.95 | - |
| $\log_{10}\lambda_{E}^{*}$ (log d^-2^) | -0.29 (7.92) | 0.01 (76.3) |
| $d_{I}$ (d^-1^) | 0.1 | - |
| $d_{D}$ (d^-1^) | 0.01 (37.2) | 1.00 (33.3) |
| $\gamma$ (cells^-1^) | 7.42×10^4^ (37.4) | 1.35 (20.9) |
| $\alpha_{E}$ (d^-1^) | 0.62 (3.6) | 0.09 (22.5) |
| $\theta_{E}$ (cells mL^-1^) | 0.10 | - |
| $d_{E}$ (d^-1^) | 1.00 | - |
| $\log_{10}T(0)$ (log cells mL^-1^) | 3.38 (1.75) | 0.01 (107) |

**Table S12:** **Population parameter estimates for model #10.** The fixed and random effects of each parameter are provided along with respective percent standard errors in parentheses. In addition to the parameters fixed in the best-fit model, $f_{D}$ is fixed to 0.95 [1].

**References**

1. Wang S, Hottz P, Schechter M, Rong L. Modeling the Slow CD4+ T Cell Decline in HIV-Infected Individuals. PLoS Comput Biol. 2015;11(12):e1004665. Epub 20151228. doi: 10.1371/journal.pcbi.1004665. PubMed PMID: 26709961; PubMed Central PMCID: PMCPMC4692447.
