# Supplementary material for: Antiviral capacity of the early CD8 T-cell response is predictive of natural control of SIV infection: Learning in vivo dynamics using ex vivo data
Source: PLoS Comput Biol. 2024 Sep 10;20(9):e1012434. doi: 10.1371/journal.pcbi.1012434 (PMC11414924; doi:10.1371/journal.pcbi.1012434)
Supplement: S14 Table — Contribution of suppressive capacity data to BIC of model #1 was removed to compare it with models #10 and #11. (DOCX) [file pcbi.1012434.s035.docx]

| **Model** | **Description** | **BIC** |
| --- | --- | --- |
| 1 | Main model | 972 |
| 10 | Main model fitted with constant $k$ without the suppressive capacity data | 1168 |
| 11 | Main model fitted without the suppressive capacity data | 1022 |

**Table S14:** **Comparison of model fits without suppressive capacity data.** Contribution of suppressive capacity data to BIC of model #1 was removed to compare it with models #10 and #11.
