# Supplementary material for: Antiviral capacity of the early CD8 T-cell response is predictive of natural control of SIV infection: Learning in vivo dynamics using ex vivo data
Source: PLoS Comput Biol. 2024 Sep 10;20(9):e1012434. doi: 10.1371/journal.pcbi.1012434 (PMC11414924; doi:10.1371/journal.pcbi.1012434)
Supplement: S15 Table — The fixed and random effects of each parameter is provided along with respective percent standard errors in parentheses. (DOCX) [file pcbi.1012434.s036.docx]

| **Parameter (Units)** | **Fixed effect** | **Random effect** |
| --- | --- | --- |
| $\lambda$ (cells mL^-1^ d^-1^) | 22.2 (18.7) | 0.62 (34.5) |
| $\log_{10}\beta'$ (log mL cells^-1^ d^-1^) | -4.19 (1.9) | 0.02 (286) |
| $\log_{10}\lambda_{E}^{*}$ (log d^-2^) | -3.85 (8.34) | 0.34 (25.8) |
| $d_{I}$ (d^-1^) | 0.10 | - |
| $\gamma$ (cells^-1^) | 2.77 (42.5) | 1.56 (19.6) |
| $\alpha_{E}$ (d^-1^) | 1.75×10^-11^ (523) | 2.84 (323) |
| $\theta_{E}$ (cells mL^-1^) | 0.10 | - |
| $d_{E}$ (d^-1^) | 1.00 | - |
| $\log_{10}T(0)$ (log cells mL^-1^) | 4.41 (1.82) | 0.02 (26.8) |

**Table S15:** **Population parameter estimates for model #12.** The fixed and random effects of each parameter is provided along with respective percent standard errors in parentheses.
