# Supplementary material for: Antiviral capacity of the early CD8 T-cell response is predictive of natural control of SIV infection: Learning in vivo dynamics using ex vivo data
Source: PLoS Comput Biol. 2024 Sep 10;20(9):e1012434. doi: 10.1371/journal.pcbi.1012434 (PMC11414924; doi:10.1371/journal.pcbi.1012434)
Supplement: S16 Table — Note that the exhaustion compartment, Q, is present only in models #3 and #4. Viral inoculum sizes have been estimated using the volumes of distribution [1]. (DOCX) [file pcbi.1012434.s037.docx]

| **Variable** | **Description** | **Initial value** |
| --- | --- | --- |
| $T(0)$ | Initial concentration of target CD4 T-cells | $\lambda/d_{T}$ |
| $I(0)$ | Initial concentration of productively infected cells | 0 |
| $D(0)$ | Initial concentration of non-productively infected cells | 0 |
| $V(0)$ | Viral inoculum size | 10^-2.76^ copies mL^-1^ for macaques BC094, BC179, BD536, and BO186  10^-1.76^ copies mL^-1^ for the other macaques |
| $E(0)$ | Initial concentration of SIV-specific effector CD8 T-cells | 0 |
| $k(0)$ | Killing rate constant of productively infected cells by virus-specific CD8 T-cells | 0 |
| $Q(0)$ | Initial exhaustion level | 0 |

**Table S16:** **Initial conditions used for in vivo model fitting.** Note that the exhaustion compartment, $Q$, is present only in models #3 and #4. Viral inoculum sizes have been estimated using the volumes of distribution [1].

**References**

1. Passaes C, Millet A, Madelain V, Monceaux V, David A, Versmisse P, et al. Optimal maturation of the SIV-specific CD8(+) T cell response after primary infection is associated with natural control of SIV: ANRS SIC study. Cell Rep. 2020;32(12):108174. doi: 10.1016/j.celrep.2020.108174. PubMed PMID: 32966788.
